# Graph–Tabular Latent Fusion for Non-Contact Body Temperature Prediction from Thermal Facial Landmarks

**DOI:** 10.3390/s26113619

**Published:** 2026-06-05

**Authors:** Yean Chun Ng, Alexander G. Belyaev, Florence C. M. Choong, Shahrel Azmin Suandi, Joon Huang Chuah, Bhuvendhraa Rudrusamy

**Affiliations:** 1 School of Engineering and Physical Sciences, Heriot-Watt University Malaysia, Putrajaya 62200, Malaysia; y.ng@hw.ac.uk (Y.C.N.); 2 School of Engineering and Physical Sciences, Heriot-Watt University, Edinburgh EH14 4AS, UK; 3 School of Electrical and Electronic Engineering, Universiti Sains Malaysia, Nibong Tebal 14300, Malaysia; 4 Department of Electrical Engineering, Faculty of Engineering, University of Malaya, Kuala Lumpur 50603, Malaysia

**Keywords:** thermal imaging, body temperature prediction, facial landmarks, graph neural networks, graph–tabular fusion, latent representation learning, missing data, imputation

## Abstract

Non-contact body-temperature prediction from facial thermography is affected by pose, occlusion, missing measurements, and inter-subject variation. This study proposes a graph–tabular latent-representation fusion framework for predicting body temperature from thermal facial landmark profiles. A Pearson correlation coefficient (PCC)-guided landmark graph models landmark-to-landmark thermal dependencies. At the same time, the same landmark-temperature signal is retained as a tabular representation to preserve global temperature-pattern interactions. The graph and tabular branches are encoded independently, fused at the latent level, and trained for target-landmark temperature regression with auxiliary reconstruction losses. Experiments were conducted on TFD68 under complete, missing completely at random (MCAR), and structured missing not at random (MNAR) conditions. The structured MNAR simulation combines 3D head-pose visibility modelling, accessory-driven occlusion, validation against real TFD68 occlusion annotations, and graph-construction sensitivity analyses. Results show that selected fused configurations improve over strong stand-alone graph and tabular baselines, particularly under MNAR-imputed evaluation, with the best selected configuration reducing prediction error by approximately 6%. Statistical testing further confirms significant improvements in most MNAR fused–baseline comparisons. Accuracy–efficiency analysis shows that fusion improves robustness at the cost of additional inference time, providing a flexible design space for thermal landmark-based body-temperature prediction.

## 1. Introduction

Body temperature is an important physiological indicator for health monitoring, clinical assessment, and early detection of abnormal physiological states. Conventional body temperature measurement methods, such as oral, tympanic, axillary, or contact-based sensors, can provide reliable readings. Still, they often require direct contact, controlled measurement procedures, or repeated manual acquisition. Thermal imaging offers a non-contact alternative by capturing facial surface-temperature distributions, making it attractive for rapid and unobtrusive body temperature estimation. Thermal facial analysis has also been explored beyond screening applications; for example, Baskaran et al. investigated facial landmark detection on thermal images as a prognostic tool in emergency department settings, highlighting the potential clinical relevance of landmark-based thermal information [1]. Nevertheless, predicting body temperature from facial thermal patterns remains challenging because facial temperature is not uniformly distributed and can be affected by anatomical location, local blood perfusion, environmental conditions, individual physiological variation, and partial missingness or occlusion.

A major practical source of difficulty is that missing landmark temperatures do not usually occur uniformly at random. In real thermal facial screening, missingness is often caused by structured factors such as head pose, landmark location, glasses, and face masks. Large yaw or pitch angles can produce self-occlusion of contour-side landmarks, glasses can obscure periocular landmarks, and face masks can hide the nose, mouth, and lower-face regions. Such missingness is better described as MNAR rather than MCAR, because the probability of a landmark being missing depends on the acquisition condition and the anatomical location of the landmark [2,3]. Therefore, evaluating body-temperature prediction only under complete data or uniformly random missingness may overestimate robustness in realistic screening conditions. To address this issue, this study evaluates models under structured MNAR missingness generated from 3D head-pose visibility modelling and accessory-specific occlusion patterns, with the resulting landmark-wise missingness distributions validated against real TFD68 occlusion annotations [4].

Beyond missingness, facial thermal images also contain structured spatial information. Different facial landmarks, such as regions around the forehead, eyes, nose, cheeks, and mouth, may exhibit distinct yet correlated temperature patterns. These inter-landmark dependencies suggest that body temperature prediction should not rely only on isolated landmark values, but should also consider relationships among landmarks. A graph-based representation is well-suited for this purpose because landmarks can be represented as nodes and their thermal dependencies as edges. Graph neural networks (GNNs) can then learn relational representations through message passing, allowing each landmark to incorporate information from connected landmarks. This relational inductive bias is effective for modelling structured data and node dependencies [5,6,7].

Despite the advantages of graph-based learning, graph representations alone may not fully capture global feature interactions present in the original thermal profile. Graph connectivity imposes a structural prior that emphasises relational dependencies, but some predictive patterns may exist across the complete set of landmark measurements without being explicitly represented by graph edges. Tabular representations provide a complementary view by preserving the landmark temperature profile as a fixed-length feature vector. Dedicated tabular learning architectures, including multilayer perceptrons, residual MLPs, attentive models, and transformer-based encoders, have been widely studied for capturing feature interactions in structured data [8,9,10,11]. Therefore, combining graph-based and tabular-based representations can provide a more complete modelling framework for body temperature prediction.

In this work, we propose a graph–tabular latent-representation fusion framework for body temperature prediction from thermal facial landmark data. The proposed method uses two complementary branches. The graph branch models landmark-level thermal dependencies using a graph neural network backbone. In contrast, the tabular branch learns global feature interactions from a fixed-length representation of the same sample. The two branches are encoded independently to obtain branch-specific latent representations, which are then fused at the latent level and passed to a fully connected prediction head for target-node regression. This design allows the model to combine relational reasoning from the graph branch with global abstraction from the tabular branch.

To construct the graph representation, we first analyse inter-landmark thermal relationships using Pearson, Spearman, and Kendall correlation measures. The high visual and structural agreement among these correlation matrices indicates that landmark temperature dependencies are largely consistent across correlation criteria. Based on this analysis, PCC is selected as the primary criterion for graph construction due to its interpretability, direct magnitude interpretation, and suitability for defining a stable correlation-guided structural prior. A shared graph topology is constructed by connecting landmark pairs whose PCC exceeds a predefined threshold, with subject-specific temperature values serving as node features. This results in a multi-graph formulation in which each subject is represented by an individual landmark-temperature graph with a common structural prior.

The proposed architecture further incorporates auxiliary reconstruction objectives for both graph and tabular branches. These auxiliary decoders encourage each latent representation to preserve broader sample-level information rather than collapsing into a narrow representation optimised only for the final scalar prediction. The final objective jointly optimises the fused prediction loss and the branch-specific reconstruction losses, enabling the model to learn informative and complementary latent spaces.

Unlike existing graph–tabular fusion approaches that rely on heterogeneous modalities or auxiliary descriptors, this work derives complementary graph and tabular representations from a single thermal facial landmark signal. The proposed framework jointly exploits relational inductive bias through graph modelling and global feature abstraction through tabular learning, enabling a unified and principled latent-level fusion strategy for body-temperature prediction. The main contributions of this study are summarised as follows:

- We propose a graph–tabular latent-representation fusion framework that derives complementary relational and global feature representations from a single thermal facial landmark signal for non-contact body temperature prediction.
- We introduce a PCC-guided facial landmark graph construction strategy that establishes a shared correlation-derived topology while preserving subject-specific thermal node features in a multi-graph formulation, supported by threshold sensitivity and correlation-method control analyses.
- We construct a structured MNAR missingness simulation based on 3D head-pose visibility modelling and accessory-specific occlusion scenarios, and validate its landmark-wise missingness patterns against real TFD68 occlusion annotations.
- We conduct a comprehensive evaluation under occlusion-driven MNAR and MCAR missingness, demonstrating that latent-level graph–tabular fusion improves robustness over strong stand-alone graph and tabular baselines.
- We analyse accuracy–efficiency trade-offs by jointly reporting prediction error, inference time, and throughput, quantifying the deployment cost associated with improved robustness under missing and imputed landmark conditions.

Overall, this study demonstrates that combining graph-structured relational modelling with tabular feature abstraction provides a principled and flexible framework for thermal landmark-based body temperature prediction. The remainder of this paper is organised as follows. Section 2 reviews related work on non-contact thermal temperature estimation, conventional and deep learning approaches, graph-based relational learning, graph construction strategies, and graph–tabular fusion. Section 3 presents the proposed methodology, including PCC-guided graph construction and the graph–tabular latent-representation fusion model. Section 4 describes the experimental setup, including dataset preparation, protocols for missingness and imputation, baseline references, fusion-model configurations, and evaluation metrics. Section 5 reports and discusses the experimental results for both baseline and fusion models, including statistical analysis. Finally, Section 6 concludes the paper and outlines future research directions. The dataset, source code, and pre-trained models are publicly available at Github (https://github.com/lucas-nyc/GTLF_BTP (accessed on 1 May 2026)).

## 2. Related Work

Fever is a hallmark symptom of many infectious diseases (including COVID-19), making body temperature screening a critical tool for outbreak prevention [12]. During the COVID-19 pandemic, health authorities widely recommended non-contact screening at public venues (airports, hospitals, workplaces) to identify potential cases early, and infrared thermography (IRT) quickly became a preferred non-invasive modality for mass triage [13,14]. However, numerous evaluations have shown that uncalibrated thermal screening can suffer from poor sensitivity and specificity in uncontrolled settings; for example, broad airport screening during past outbreaks sometimes detected few true cases, highlighting the need for more robust algorithmic correction and modelling [13]. Infrared thermography remains attractive because it produces per-pixel temperature maps that can be sampled at physiological landmarks such as the inner canthi, temples, and forehead, with the inner canthus frequently reported as one of the regions most correlated with core temperature [15,16]. Typical operational pipelines either fuse an RGB camera with a thermal sensor (to exploit reliable RGB face detection and map results to the thermal frame) or operate directly on thermal frames using learned detectors and regressors [4,17]. Curated datasets such as TFD68, annotated with per-pixel temperature and designed to be pose- and occlusion-invariant, have accelerated work that leverages thermographic cues as complementary biometric or diagnostic features [4]. Likewise, recent studies explicitly exploit per-pixel temperature to detect occluded facial landmarks across poses and mask occlusions, reducing landmark mislocalisation that would otherwise corrupt temperature mapping and downstream estimation [18].

### 2.1. Conventional Machine-Learning Approaches

Traditional non-contact temperature estimation methods commonly rely on engineered thermal region of interest (ROI) statistics and conventional regression models, including linear or multivariate regression, support vector regression, decision tree ensembles, random forests, and shallow neural models [12]. For example, a recent literature found that a linear model achieved the lowest root mean squared error (RMSE) among several regression models using facial thermal ROIs, showing that simple predictors can remain competitive when ROI extraction and measurement conditions are controlled [12]. This motivates the inclusion of a simple linear-projection baseline in this work. At the same time, an MLP baseline is also included because stacked dense transformations can capture non-linear interactions among compact, continuous landmark-temperature features and have been shown to remain competitive for tabular learning [8,9]. More broadly, fever-screening performance is strongly influenced by ROI selection, environmental control, and sensor calibration [19,20,21], and some studies have incorporated multivariate regression, variance-based fusion, or Kalman filtering to improve non-contact temperature estimation [22]. However, these conventional approaches depend heavily on manually selected inputs and model spatial or relational context only weakly, making them sensitive to pose changes, occlusion, and inter-subject variability.

### 2.2. Deep Learning Approaches

Deep learning approaches replace manual feature engineering with representations learned directly from raw images or structured covariates. In thermal imaging, convolutional neural network (CNN) backbones and multi-task architectures have been widely adopted for landmark localisation and related preprocessing tasks, improving robustness under modality-specific challenges such as low texture, noise, and occlusion [18,23,24]. Beyond preprocessing, recent studies report that deep regression models can outperform classical approaches when sufficiently large and diverse annotated thermal datasets are available [4,25]. Consequently, deep networks have become the dominant solution for upstream tasks, including thermal face detection, landmark localisation, and mask detection, all of which are essential for reliable temperature sampling in practical deployment [17,25]. For structured, non-image inputs, fully connected architectures remain strong and competitive baselines. Notably, [8] demonstrated that, under careful training and hyperparameter tuning, simple MLPs, particularly ResNet-style MLPs with residual connections can achieve performance comparable to or exceeding more complex tabular models. This insight is directly relevant to thermal-screening pipelines that incorporate scalar covariates or ROI temperature features, as it supports using lightweight tabular branches rather than assuming convolutional architectures are necessary for all inputs. Despite these advantages, single-branch deep models typically process each sample independently and do not explicitly account for relational dependencies unless additional structure is introduced.

### 2.3. Graph-Based Models and Relational Learning

GNNs provide a principled framework for modelling relational context, such as spatial, temporal, or structural, in prediction tasks by explicitly encoding dependencies among samples, sensors, or regions of interest. Empirical studies demonstrate that Graph Convolutional Networks (GCNs) are highly effective for fusing information in non-Euclidean domains, such as sensor networks, where spatial or relational dependencies are critical [26]. In temperature and environmental forecasting, graph-based models, including diffusion-based approaches such as DCRNN and adaptive-topology models such as Graph WaveNet, have shown improved predictive performance by explicitly modelling spatial proximity and latent interactions between nodes [27,28,29].

Inductive architectures such as GraphSAGE aggregate neighbourhood information to learn node embeddings that generalise to unseen nodes, making them particularly suitable for dynamic scenarios where the underlying graph evolves [7]. Building on this, Graph Attention Networks (GATs) introduce learnable attention mechanisms to weight neighbouring nodes based on their relevance, enabling the model to suppress noisy or less informative signals [30], while Graph Isomorphism Networks (GINs) enhance representational power through injective aggregation schemes that approach the discriminative capability of the Weisfeiler–Lehman graph isomorphism test [31]. Although direct applications of GNNs to thermal-fever screening remain limited, insights from these domains suggest that constructing graphs over individuals, ROIs, or sensors can enable the propagation of reliable contextual information, thereby improving robustness in the presence of noise, occlusion, or missing measurements.

### 2.4. Graph Construction Strategies for Temperature Inference

A key step in any graph-based method is graph construction: deciding which entities to treat as nodes and how to connect them. Typical strategies connect nodes by spatial proximity (distance thresholds or k-nearest neighbours), feature similarity (embedding distances), or domain-informed adjacency (anatomical adjacency among facial ROI or social-contact links among people). Multi-scale or hierarchical graphs can capture both local and global factors affecting temperature readings. Learnable adjacency modules: small neural networks that predict edge weights from node features have also been proposed to dynamically adapt the topology to the data [26]. In geographical applications, an “adaptive graph creation” has been used to capture distance-based similarity between locations [32]. Previous literature presents a method that automatically creates graph edges reflecting geographic proximity, thereby improving clustering and the prediction of spatial phenomena [32]. Analogously, for thermal imaging, one could use the distance between face bounding boxes or feature distances in embedding space to define graph edges. These graph construction choices matter: a node whose face is partly masked can borrow context from neighbouring body regions or proximate individuals, yielding more robust estimates.

### 2.5. Graph and Tabular Learning Fusion

Recent work increasingly combines relational encoders with tabular or descriptor-based branches, rather than forcing all information into a single representation. Rao et al. proposed a fusion model for chronic kidney disease prediction in which a GNN branch and a tabular deep-learning branch were trained separately and then fused for the final prediction, with the joint model outperforming either branch alone [33]. In tabular-data learning, TabGNN constructs multiplex graphs over samples, learns graph embeddings, and concatenates those learned embeddings with the original tabular representations before passing them to downstream predictors [34]. In molecular property prediction, FP-GNN combines a graph-attention-based molecular encoder with fingerprint descriptors and reports that the two modalities are complementary rather than redundant [35]. Related work on contextual graph embeddings for tabular data also shows that graph-style interaction modelling can improve representation quality when conventional tabular learning ignores relations among features or records [36].

A related representation-learning idea is also seen in the Graph Unseen Node Embedding Learning Network proposed by Xu et al. [37] for drug–target affinity prediction. Their work is not a graph–tabular fusion model, nor does it construct two complementary views from the same dataset in the same way as the present study. However, it shares a broader conceptual similarity: graph-based representation learning is used to obtain informative latent embeddings that improve downstream prediction. This supports the general premise that learned graph representations can provide useful predictive structure beyond raw input features alone.

Taken together, these studies support dual-branch or fusion designs in which graph-structured and non-graph features are first encoded separately and then combined at a higher representation level for the final regression or classification layer. In the present work, this principle is applied to thermal facial landmark analysis by learning a graph-side latent representation from the landmark dependency structure and a tabular-side latent representation from the landmark-temperature vector, and then fusing them for body temperature prediction.

### 2.6. Synthesis and Research Gap

The reviewed literature can be organised into four methodological streams, each contributing a distinct component to non-contact body temperature estimation and representation learning. In thermal screening, Nguyen et al. [19] compared multiple infrared thermal detection systems (ITDSs) for mass fever screening, demonstrating the practical feasibility of rapid, non-contact screening while showing that performance depends on device configuration and screening conditions. Tay et al. [20] further evaluated ITDS deployment in a tropical healthcare setting, highlighting the influence of ambient conditions and calibration on the reliability of fever screening. More recently, Limpabandhu et al. [12] moved beyond threshold-based screening by using facial thermal regions as regression inputs for core body temperature prediction, showing that selected facial ROIs, particularly the temple and nose regions, contain useful predictive information. Collectively, these studies establish the value of facial thermal cues for body temperature estimation, but they remain largely ROI-driven and do not explicitly model dependencies among facial landmarks.

For compact structured inputs, Gorishniy et al. [8] showed that carefully tuned MLPs and ResNet-style tabular networks are strong baselines for tabular deep learning. This contribution is relevant because landmark-temperature samples can naturally be represented as fixed-length tabular vectors. However, tabular encoders mainly learn global feature interactions from the landmark-temperature vector and do not explicitly encode anatomical or statistical relationships between individual landmarks.

Graph neural networks provide a complementary relational perspective. Graph-SAGE [7] introduced an inductive neighbourhood-aggregation framework, making it suitable for learning node representations when each facial landmark is treated as a graph node. GAT [30] extended graph aggregation with attention, allowing the model to assign different importance weights to neighbouring landmarks. GINs [31] provided a more expressive aggregation mechanism motivated by graph-isomorphism theory, making it a strong reference architecture for evaluating whether graph structure contributes to prediction. These graph models motivate the use of landmark-to-landmark message passing, but they were not developed specifically for thermal body temperature prediction.

Graph–tabular and multi-view fusion studies further suggest that relational and non-relational representations can be complementary. Rao et al. [33] fused a GNN branch with a tabular branch for chronic kidney disease prediction, showing that graph-based and tabular clinical representations can be encoded separately and combined at a latent level. Guo et al. [34] proposed TabGNN, which constructs multiplex graphs to model sample relations and then combines learned graph embeddings with tabular representations. Cai et al. [35] introduced FP-GNN for molecular property prediction, combining molecular graph representations with fingerprint descriptors and showing that the two views provide complementary information. Villaizán-Vallelado et al. [36] used an Interaction Network to produce contextual embeddings for tabular features, demonstrating another way to inject relational inductive bias into tabular prediction. Zhao et al. [37] further showed that molecular property prediction can benefit from combining intra-molecule graph representations with inter-molecule similarity-graph structure. These studies support latent-level fusion, but they mainly address clinical tabular prediction, general tabular prediction, or molecular property estimation, in which graph and tabular descriptors are often naturally distinct or externally defined.

Although these fusion studies demonstrate the general value of combining relational and non-relational representations, they are not directly adapted to thermal facial body temperature prediction. Existing graph–tabular fusion methods usually assume that the graph and tabular branches originate from naturally heterogeneous sources, such as clinical variables and patient graphs, molecular structures and fingerprint descriptors, or sample-relation graphs and tabular attributes. Thermal facial landmark data have different characteristics. First, the input is a single thermal signal sampled over facial landmarks rather than multiple heterogeneous modalities. Second, the data are affected by occlusion-driven missingness due to head pose, glasses, and face masks, leading to high, non-uniform missing rates across landmarks. Third, facial temperature distributions vary substantially across individuals because of anatomical, physiological, environmental, and acquisition-related factors. Finally, temperature is not uniformly distributed across the face; some landmarks are more informative or more stable than others, and their relationships may depend on anatomical proximity or statistical thermal dependency. These properties make it insufficient to transfer existing graph–tabular fusion designs directly without adapting the representation strategy to thermal landmark-temperature data.

Despite these advances, existing thermal-screening studies have rarely investigated graph–tabular latent-representation fusion for body temperature prediction from facial landmark temperatures. Existing thermal studies primarily rely on ROI selection, device-level thermal screening, image preprocessing, landmark localisation, or conventional regression, while existing graph–tabular fusion studies focus mostly on domains where relational and tabular views are derived from different modalities, sample-level relations, molecular graphs, or handcrafted descriptors. In contrast, the present problem requires extracting two complementary representations from the same thermal landmark-temperature sample: a graph view that captures landmark-to-landmark thermal dependencies and a tabular view that preserves the global temperature pattern across all landmarks. This defines the specific research gap addressed in this study: no prior work has systematically constructed and fused graph-side and tabular-side latent representations from a single thermal facial landmark signal to predict body temperature under occlusion-driven missingness robustly. The proposed framework is, therefore, necessary to jointly exploit local relational structure, global landmark-temperature interactions, and the missingness characteristics of thermal facial data.

## 3. Methodology

The proposed methodology uses TFD68, a thermal facial dataset containing facial thermal images, 68 annotated facial landmarks, and corresponding landmark-temperature profiles. In this study, each sample is represented by the temperature values extracted at the annotated facial landmarks. At the same time, the full details of the dataset acquisition, annotation protocol, and data splits are discussed further in Section 4.1.

**Figure 1:**
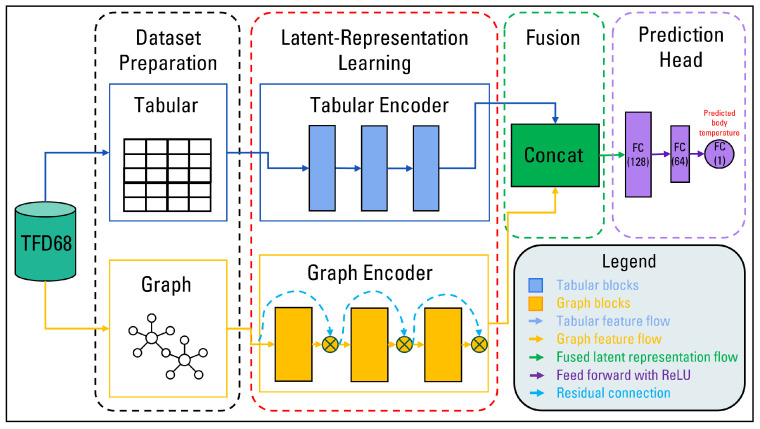
Overview of the proposed graph–tabular latent-representation fusion framework for body temperature prediction. TFD68 landmark-temperature data are represented as two complementary inputs: a tabular view and a graph view. The tabular branch encodes the global landmark-temperature vector, while the graph branch encodes landmark-to-landmark thermal dependencies through graph blocks with residual connections. Blue blocks and blue arrows denote tabular blocks and tabular feature flow, respectively, while yellow blocks and yellow arrows denote graph blocks and graph feature flow. Cyan dashed arrows indicate residual connections within the graph branch. The green block labelled “Concat” denotes latent-level fusion of the independently encoded tabular and graph embeddings, and the green arrow denotes the fused latent embedding flows into the prediction head. In the prediction head, the labelled fully connected layers map the fused representation through FC (128), FC (64), and FC (1), where purple arrows denote ReLU-activated prediction-head flow. The final FC (1) output represents the predicted body temperature.

The methodology consists of two main stages. First, facial landmark-temperature profiles from TFD68 are used to construct subject-specific landmark graphs, where each node represents a facial landmark and each edge encodes the thermal dependency structure derived from inter-landmark correlations. Second, a graph–tabular latent-representation fusion model is introduced for body temperature prediction. As illustrated in Figure 1, the same landmark-temperature sample is represented through two complementary views: a tabular view that preserves the global landmark-temperature vector, and a graph view that captures relational dependencies among landmarks through message passing. The tabular and graph branches are encoded independently into latent representations, which are then concatenated and passed to a fully connected (FC) prediction head for final body temperature estimation. In this design, the tabular branch captures global temperature-pattern interactions, while the graph branch captures local landmark-to-landmark thermal dependencies.

### 3.1. Graph Construction

To analyse spatial–thermal relationships among facial landmarks, we construct a landmark graph in which each node represents one facial landmark and each edge represents a strong thermal dependency between two landmarks. The graph is not intended to encode facial geometry directly. Instead, it defines a data-driven structural prior that captures how landmark temperatures co-vary across subjects. This shared topology is then used across all subjects, while each subject retains its own landmark-temperature values as node features.

**Figure 2:**
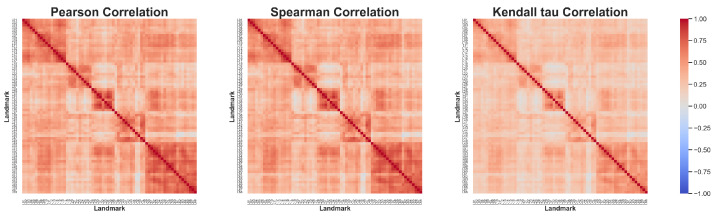
Correlation heatmaps computed from the landmark-temperature matrix $X\in R^{S\times 68}$ using Pearson correlation, Spearman rank correlation, and Kendall’s tau. Each heatmap is a 68×68 matrix in which entry (i,j) represents the thermal association between landmarks *i* and *j* across all samples. Diagonal entries indicate landmark self-correlation, while off-diagonal entries indicate inter-landmark thermal dependencies. Similar block structures across the three heatmaps suggest that the global landmark dependency pattern is preserved across correlation measures.

The landmark-temperature data are first arranged into a matrix $X\in R^{S\times 68}$, where *S* denotes the number of samples and each column $x_{i}$ contains the temperature values of landmark *i* across all samples. To examine whether the inter-landmark dependency structure is stable across correlation definitions, three correlation matrices are computed from X using Pearson correlation [38], Spearman rank correlation [39], and Kendall’s tau [40]. For a given correlation method *m*, the resulting matrix is denoted as $R^{\left(m\right)}\in R^{68\times 68}$, where each entry $r_{ij}^{\left(m\right)}$ measures the thermal association between landmarks *i* and *j* across the dataset. Therefore, each heatmap in Figure 2 visualises one complete 68×68 landmark-to-landmark dependency matrix.

As shown in Figure 2, the three correlation methods produce broadly similar global dependency patterns across the 68 landmarks. The strong diagonal values correspond to the expected self-correlation of each landmark, while the off-diagonal entries reveal groups of landmarks with moderate to strong thermal associations. Pearson and Spearman correlations exhibit especially similar heatmap structures, suggesting that the dominant inter-landmark relationships are largely monotonic and approximately linear. Kendall’s tau produces a visually similar structure but with reduced magnitude, which is expected because it is generally more conservative in scale. Overall, the heatmaps indicate that the major landmark dependency structure is preserved across correlation measures.

In addition to visual structural agreement, the numerical deviation between correlation matrices is evaluated using the Frobenius norm. For two correlation methods *a* and *b*, the Frobenius difference is computed as

(1) $$D\left(a,b\right)={∥R^{\left(a\right)}-R^{\left(b\right)}∥}_{F}=\sqrt{\sum_{i=1}^{68}\sum_{j=1}^{68}{\left(r_{ij}^{\left(a\right)}-r_{ij}^{\left(b\right)}\right)}^{2}}$$

**Figure 3:**
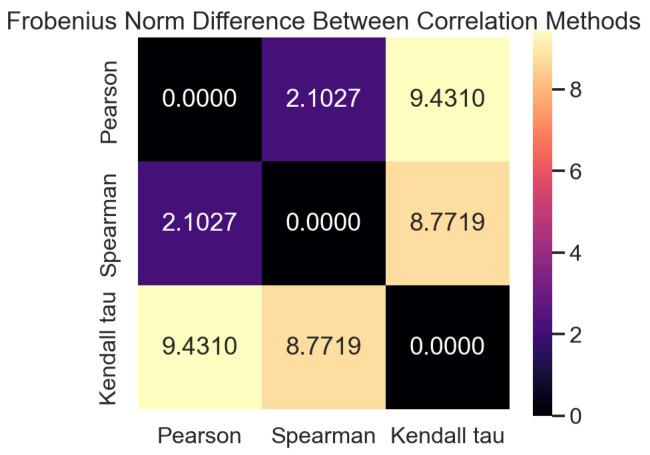
Frobenius difference between correlation methods. Each entry compares two complete 68×68 landmark correlation matrices. Lower values indicate smaller numerical deviation between correlation structures.

Thus, each entry in Figure 3 is obtained by comparing two complete 68×68 correlation matrices from Figure 2. The zero-valued diagonal confirms that each matrix is identical to itself. The Pearson–Spearman difference is low (2.1027), supporting the visual observation that both methods produce very similar correlation structures. Larger differences are observed for Kendall’s tau, namely Pearson–Kendall (9.4310) and Spearman–Kendall (8.7719). These larger values do not necessarily indicate a different dependency structure; rather, they mainly reflect the lower numerical scale commonly produced by Kendall’s tau. Therefore, the Frobenius analysis suggests that Pearson and Spearman are numerically closest, while Kendall’s tau remains structurally similar despite magnitude differences.

Based on this analysis, PCC is used as the primary criterion for graph construction. This choice is motivated by three considerations. First, landmark temperatures are continuous measurements, making Pearson correlation a direct and interpretable measure of linear thermal co-variation between landmark pairs. Second, the heatmap and Frobenius analyses show that PCC captures a dependency structure that is highly consistent with the rank-based alternatives, especially Spearman correlation. Third, PCC values have a direct magnitude interpretation, which makes threshold-based edge selection straightforward and transparent. The use of PCC is, therefore, appropriate for defining a stable and interpretable structural prior. The purpose of this prior is not to fully model the downstream nonlinear prediction function, but to identify thermally related landmarks over which the graph neural network can subsequently learn nonlinear transformations.

Let $T\in R^{S\times N}$ denote the landmark-temperature matrix, where *S* is the number of subjects and N=68 is the number of landmarks. The pairwise Pearson correlation between landmarks *i* and *j* is computed across all subjects. Following common interpretations of correlation strength [41], values in the range [0.6,1.0] are considered strong positive associations. Therefore, the graph threshold is set to τ=0.6 to retain strongly correlated landmark pairs while avoiding an overly dense topology. The robustness of this threshold choice, together with a control experiment comparing PCC, Spearman, and Kendall graph topologies, is evaluated later in Section 5.3.

Each graph is defined as G=(V,E), where each node i∈V corresponds to one facial landmark, and its associated feature is the subject-specific temperature value. An edge (i,j)∈E exists if:

(2) PCC(i,j)>τ

where τ=0.6.

This formulation captures intrinsic thermal dependencies between landmarks while maintaining interpretability. Constructing a single graph with averaged node features across all subjects is not appropriate because averaging would remove subject-specific thermal variation. Therefore, a multi-graph formulation is adopted. The graph topology, or edge structure, is shared across subjects based on the PCC-derived structural prior, while node features are instantiated separately using subject-specific landmark temperature values. This allows each graph to preserve individual thermal profiles while maintaining a consistent relational structure across the dataset. The graph construction overview is shown in Algorithm 1.

This process yields a collection of landmark-temperature graphs, each capturing subject-specific thermal variation within a shared correlation-derived topology. Such a representation is well-suited to graph-based learning methods because it enables localised thermal dependencies to be modelled during downstream prediction while preserving individual differences in facial temperature profiles.
**Algorithm 1** PCC-Based Landmark Graph Construction**Require:** $T\in R^{S\times N}$; τ=0.6**Ensure:** ${\left\{G_{s}\right\}}_{s=1}^{S}$  1:Initialise: E←∅  2:**for** i=1 to *N* **do**  3:      **for** j=i+1 to *N* **do**  4:            $\rho_{ij}\leftarrow \mathrm{corr}\left(T_{:,i},T_{:,j}\right)$  5:            **if** $\rho_{ij}>\tau$ **then**  6:                 E←E∪{(i,j),(j,i)}  7:            **end if**  8:      **end for**  9:**end for**10:**for** s=1 to *S* **do**11:      V←{1,…,N}12:      $x_{s,i}\leftarrow T_{s,i},\;\forall i\in V$13:      $G_{s}\leftarrow \left(V,E,{\left\{x_{s,i}\right\}}_{i=1}^{N}\right)$14:**end for**15:**return** ${\left\{G_{s}\right\}}_{s=1}^{S}$

### 3.2. Graph–Tabular Latent–Representation Fusion Model

To jointly exploit relational structure and global feature interactions, we employ a latent-level fusion regressor, denoted by $F_{\Theta }$. Although both inputs are derived from the same sample, they provide two complementary views of the data: a graph-structured view and a tabular view. The graph branch models relational dependencies among nodes or landmarks, while the tabular branch captures global feature interactions that are not explicitly constrained by graph connectivity. This design is motivated by the relational inductive bias of graph neural networks, which is well-suited for modelling node dependencies [5,6,7], and by evidence that tabular data benefits from dedicated architectures such as residual multilayer perceptrons, attentive feature selection, and transformer-based tokenisation [8,9,10,11]. Consequently, each branch first learns a modality-specific latent representation before the two views are fused for final prediction.

Let a graph represent one training sample

(3) G=(V,E)

where V={1,…,N} is the set of *N* landmark indices and E⊆V×V is the set of graph edges. Each index i∈V corresponds to the same landmark position across both the graph and tabular representations. In the graph branch, *i* denotes a graph node; in the tabular branch, the same index refers to the corresponding landmark component in the tabular vector. Each node i∈V is associated with a raw feature vector

(4) $$x_{i}\in R^{F_{x}}$$

a spatial coordinate vector

(5) $$c_{i}\in R^{F_{c}}$$

where $F_{c}=2$ for two-dimensional coordinates, and an observation indicator

(6) $$o_{i}\in \left\{0,1\right\}$$

where $o_{i}=1$ indicates that node *i* is observed and $o_{i}=0$ indicates that it is masked or missing. Let t∈V denote the target node index, and let $y_{t}\in R$ be the scalar response to be predicted. More generally, $y_{i}\in R$ denotes the scalar value associated with node *i* when it is available for supervision or reconstruction.

The complete model is parameterised by

(7) $$\Theta =\{\Theta_{g},\Theta_{\mathrm{tab}},\Theta_{f},\Theta_{d_{g}},\Theta_{d_{\mathrm{tab}}}\}$$

where $\Theta_{g}$ denotes the graph-branch parameters, $\Theta_{\mathrm{tab}}$ denotes the tabular-branch parameters, $\Theta_{f}$ denotes the fusion prediction-head parameters, and $\Theta_{d_{g}}$ and $\Theta_{d_{\mathrm{tab}}}$ denote the auxiliary decoder parameters for the graph and tabular branches, respectively. These parameters are not manually assigned as separate function weights. Instead, they are trainable neural network parameters learned from the training data by minimising the model objective via backpropagation. The relative contribution of the graph and tabular representations is, therefore, learned implicitly by the fusion prediction head after latent-level concatenation.

#### 3.2.1. Graph Branch

The graph branch first augments the raw node feature vector with spatial and observation information:

(8) $${\tilde{x}}_{i}=[x_{i}\;∥\;c_{i}\;∥\;o_{i}]\in R^{F_{x}+F_{c}+1}$$

where ∥ denotes vector concatenation. This augmentation enables the model to distinguish between raw node measurements, spatial locations, and observation statuses. The augmented node feature matrix is denoted as

(9) $$\tilde{X}={\left[{\tilde{x}}_{1},\ldots ,{\tilde{x}}_{N}\right]}^{⊤}\in R^{N\times (F_{x}+F_{c}+1)}$$

Before applying the stacked message-passing layers, the graph encoder maps each augmented node input into a shared hidden space:

(10) $$h_{i}^{\left(0\right)}=\mathrm{Drop}\left(\sigma_{\mathrm{act}}\left(\mathrm{BN}\left(W_{\mathrm{in}}{\tilde{x}}_{i}+b_{\mathrm{in}}\right)\right)\right)$$

where $W_{\mathrm{in}}\in R^{D_{h}\times \left(F_{x}+F_{c}+1\right)}$ and $b_{\mathrm{in}}\in R^{D_{h}}$ are learnable parameters, $D_{h}$ is the graph hidden dimension, BN(·) denotes batch normalisation, $\sigma_{\mathrm{act}}\left(\cdot \right)$ is the ReLU activation function, and Drop(·) denotes dropout. This hidden representation step is used to map heterogeneous raw inputs, namely node measurements, coordinates, and observation indicators, into a common latent space before message passing. It also allows non-linear feature interactions to be learned at the node level, keeps a shared hidden dimension across all graph layers, and supports stable residual message passing.

A configurable graph encoder then applies *L* message-passing layers, where *L* denotes the number of stacked graph layers. In the current implementation, the message-passing operator can be instantiated as GraphSAGE, GCNs, GAT, or GINs. At each layer, every node updates its representation by combining its own previous representation with information received from its neighbouring nodes. Here, *ℓ* indexes the current graph layer, and *j* is an index over the nodes whose representations are used to update node *i*. Specifically, j∈N(i)∪{i} means that the update for node *i* uses both its neighbouring nodes, denoted by N(i), and the node itself. The term $h_{j}^{\left(\ell -1\right)}$ is, therefore, the representation of node *j* from the previous layer.

(11) $${\hat{h}}_{i}^{\left(\ell \right)}=M^{\left(\ell \right)}\left(\{h_{j}^{\left(\ell -1\right)}:j\in N\left(i\right)\cup \left\{i\right\}\},E\right)\;\ell =1,\ldots ,L,$$

Equation (11) performs the message-passing step. The operator $M^{\left(\ell \right)}\left(\cdot \right)$ gathers and combines information from node *i* and its neighbours according to the selected GNN backbone. For example, the GCNs uses normalised neighbour aggregation, GraphSAGE uses neighbourhood aggregation functions, GAT applies attention weights to neighbours, and GINs use sum aggregation followed by a learnable transformation. The output ${\hat{h}}_{i}^{\left(\ell \right)}$ is the raw updated representation of node *i* before further processing.

(12) $$u_{i}^{\left(\ell \right)}=\mathrm{Drop}\left(\sigma_{\mathrm{act}}\left(\mathrm{BN}\left({\hat{h}}_{i}^{\left(\ell \right)}\right)\right)\right)$$

Equation (12) transforms this raw update into a regularised update vector $u_{i}^{\left(\ell \right)}$. BN(·) stabilises the feature distribution, the activation function $\sigma_{\mathrm{act}}\left(\cdot \right)$ introduces non-linearity, and dropout, Drop(·), reduces overfitting during training.

(13) $$h_{i}^{\left(\ell \right)}=h_{i}^{\left(\ell -1\right)}+u_{i}^{\left(\ell \right)}$$

Equation (13) applies a residual update. Instead of replacing the previous representation entirely, the model adds the new update $u_{i}^{\left(\ell \right)}$ to the previous representation $h_{i}^{\left(\ell -1\right)}$. This residual formulation allows each layer to refine the node representation while preserving useful information learned in earlier layers. After *L* graph layers, the final node embedding is

(14) $$h_{i}^{\left(L\right)}\in R^{D_{h}}.$$

For a graph with *N* nodes, the full final node-embedding matrix is

(15) $$H^{\left(L\right)}={\left[h_{1}^{\left(L\right)},\ldots ,h_{N}^{\left(L\right)}\right]}^{⊤}\in R^{N\times D_{h}}$$

Thus, the graph encoder produces one $D_{h}$-dimensional embedding per node; the number of nodes determines the number of rows in $H^{\left(L\right)}$, not the dimensionality of each node embedding. The target-node embedding is explicitly extracted as

(16) $$h_{t}=h_{t}^{\left(L\right)}\in R^{D_{h}}$$

To prevent direct target leakage, the target-node temperature is withheld from the graph input during both training and evaluation. Specifically, the raw measurement associated with node *t* is masked or replaced by the same neutral value used for missing entries, while its spatial coordinate remains available. The observation indicator is set to indicate that the target temperature is unavailable as an input. The ground-truth value $y_{t}$ is used only as the supervised prediction target. Let $O_{t}$ denote the set of observed non-target nodes:

(17) $$O_{t}=\left\{\;i\in V\;|\;i\neq t\;\mathrm{and}\;o_{i}=1\;\right\}$$

Equivalently, $O_{t}$ contains all nodes that are observed and are not the target node. The number of such nodes is $|O_{t}|$. To summarise the surrounding graph information, the model computes a context representation from the observed non-target nodes. Specifically, it first averages the final embeddings of all observed nodes except the target node:

(18) $${\bar{h}}_{\mathrm{obs}}=\frac{\sum_{i\in O_{t}}h_{i}^{\left(L\right)}}{\max\left(|O_{t}|,1\right)}$$

Here, $h_{i}^{\left(L\right)}$ denotes the final embedding of node *i* after *L* graph layers. The numerator sums the embeddings of all observed non-target nodes, while the denominator normalises this sum by the number of observed non-target nodes. The $\max(|O_{t}|,1)$ term prevents division by zero when no observed non-target node is available.

In the rare case where no observed non-target node is available, the observed-node average becomes uninformative. Therefore, the model falls back to the mean embedding of all non-target nodes:

(19) $${\bar{h}}_{\mathrm{valid}}=\frac{1}{|V_{\mathrm{valid}}|}\sum_{i\in V_{\mathrm{valid}}}h_{i}^{\left(L\right)}$$

where $V_{\mathrm{valid}}$ denotes the set of nodes that are valid for context aggregation in the current graph sample. In the current implementation, this fallback set contains the valid nodes available to the graph encoder when no observed non-target node exists. The term $|V_{\mathrm{valid}}|$ denotes the number of nodes.

The final context representation is then defined as:

(20) $${\bar{h}}_{\mathrm{ctx}}=\left\{\begin{array}{cc} {\bar{h}}_{\mathrm{obs}},\; & \mathrm{if}\;\sum_{i\in V\setminus \{t\}}o_{i}>0,\\ {\bar{h}}_{\mathrm{valid}},\; & \mathrm{otherwise} \end{array}\right.$$

Here, ${\bar{h}}_{\mathrm{ctx}}$ represents the available graph context surrounding the target node. This formulation separates target-specific information from broader contextual information: $h_{t}$ describes the target node itself, while ${\bar{h}}_{\mathrm{ctx}}$ summarises the remaining graph structure.

The target embedding and context embedding are then concatenated and projected into a compact graph-side latent representation:

(21) $$z_{g}=f_{g}\left([h_{t}\;∥\;{\bar{h}}_{\mathrm{ctx}}]\right)\in R^{D_{g}}$$

where ∥ denotes concatenation, $f_{g}\left(\cdot \right)$ is a shallow fully connected projection head, and $D_{g}$ is the graph latent dimension. In other words, $z_{g}$ is the graph branch’s compact summary of both the target node and its surrounding context.

To regularise this graph representation, the graph branch also includes an auxiliary decoder:

(22) $${\hat{y}}^{\left(g\right)}=d_{g}\left(z_{g}\right)\in R^{N}$$

where $d_{g}\left(\cdot \right)$ maps the graph latent representation to a dense node-level reconstruction vector. The *i*-th component, ${\hat{y}}_{i}^{\left(g\right)}$, denotes the graph branch’s reconstructed value for node *i*. This auxiliary reconstruction task encourages $z_{g}$ to preserve broader graph information rather than learning only the minimum required for the final target-node prediction.

#### 3.2.2. Tabular Branch

In parallel with the graph branch, the model also receives a tabular representation of the same sample. Let this tabular input be denoted as

(23) $$r={\left[r_{1},r_{2},\ldots ,r_{N}\right]}^{⊤}\in R^{N}$$

where $r_{i}$ denotes the tabular feature value associated with the *i*-th landmark position. This representation provides a non-graph view of the sample, allowing the model to learn global feature interactions without being constrained by the graph connectivity. Because the task is to predict the target node *t*, the target-associated component is removed before tabular encoding. To express this without using set subtraction, let $I_{t}$ denote the ordered set of non-target indices:

(24) $$I_{t}=\left\{\;i\in V\;|\;i\neq t\;\right\}$$

The corresponding tabular vector is then defined as

(25) $$r_{I_{t}}={\left[\;r_{i}\;\right]}_{i\in I_{t}}\in R^{N-1}$$

This removes the target value $r_{t}$ from the tabular input, preventing direct target leakage and forcing the tabular branch to infer it from the remaining measurements.

The reduced tabular vector $r_{I_{t}}$ is then processed by a tabular encoder:

(26) $$z_{\mathrm{tab}}=f_{\mathrm{tab}}\left(r_{I_{t}}\right)\in R^{D_{\mathrm{tab}}}$$

where $f_{\mathrm{tab}}\left(\cdot \right)$ denotes the tabular backbone and $D_{\mathrm{tab}}$ is the tabular latent dimension. The tabular encoder may be instantiated as an MLP, a 1D-CNN, a linear projector, a ResNet-style MLP, TabNet, or an FT-Transformer-style encoder. These alternatives allow the branch to capture different forms of feature interaction within the tabular representation. For example, residual MLPs support stable non-linear feature learning, TabNet provides attentive feature selection [10], and transformer-based tabular models learn contextualised feature interactions [8,11]. Since tabular data can exhibit heterogeneous statistical structure, no single architecture is uniformly optimal across all settings [8,9].

As in the graph branch, an auxiliary decoder is applied to the tabular latent representation:

(27) $${\hat{y}}^{\left(\mathrm{tab}\right)}=d_{\mathrm{tab}}\left(z_{\mathrm{tab}}\right)\in R^{N}$$

where $d_{\mathrm{tab}}\left(\cdot \right)$ maps the tabular latent representation to a dense node-level reconstruction vector. The *i*-th component, ${\hat{y}}_{i}^{\left(\mathrm{tab}\right)}$, denotes the tabular branch’s reconstructed value for node or landmark *i*. This auxiliary reconstruction task encourages $z_{\mathrm{tab}}$ to preserve global sample-level information rather than collapsing into a narrow task-specific code.

#### 3.2.3. Latent-Level Fusion and Prediction

After independent branch-specific encoding, the two latent representations are fused by concatenation:

(28) $$z_{f}=\left[z_{g}\;∥\;z_{\mathrm{tab}}\right]\in R^{D_{g}+D_{\mathrm{tab}}}$$

where $z_{g}$ is the graph-side latent representation and $z_{\mathrm{tab}}$ is the tabular-side latent representation.

The fused representation is then passed through a shallow fully connected prediction head:

(29) $${\hat{y}}_{t}=f_{h}\left(z_{f}\right)\in R$$

where $f_{h}\left(\cdot \right)$ maps the fused latent representation to the final scalar prediction for target node *t*.

This design is referred to as latent-level fusion because the graph and tabular modalities are first encoded independently, and their learned latent representations are combined only at a higher semantic level. This differs from decision-level fusion, where separate branch predictions are combined after independent predictions. The proposed formulation is appropriate here because the two branches exhibit different inductive biases: the graph branch emphasises relational context and local dependency structure, whereas the tabular branch emphasises global feature interactions unconstrained by graph edges. The proposed graph–tabular fusion framework is summarised in Algorithm 2.

#### 3.2.4. Training Objective

The model is trained end-to-end using a composite objective composed of one predictive loss and two auxiliary reconstruction losses. Let

(30) Ω⊆V

denote the set of nodes with valid dense reconstruction targets. The prediction loss for the target node is

(31) $$L_{\mathrm{pred}}=\ell \left({\hat{y}}_{t},y_{t}\right)$$

where ℓ(·,·) is a scalar regression loss.

The graph-branch reconstruction loss is

(32) $$L_{g}=\frac{1}{\left|\Omega \right|}\sum_{i\in \Omega }\ell \left({\hat{y}}_{i}^{\left(g\right)},y_{i}\right)$$

and the tabular-branch reconstruction loss is

(33) $$L_{\mathrm{tab}}=\frac{1}{\left|\Omega \right|}\sum_{i\in \Omega }\ell \left({\hat{y}}_{i}^{\left(\mathrm{tab}\right)},y_{i}\right)$$

The total training objective is

(34) $$L=\lambda_{f}L_{\mathrm{pred}}+\lambda_{g}L_{g}+\lambda_{\mathrm{tab}}L_{\mathrm{tab}}$$

where $\lambda_{f}$, $\lambda_{g}$, and $\lambda_{\mathrm{tab}}$ are non-negative weighting coefficients controlling the contribution of the fused prediction loss, graph reconstruction loss, and tabular reconstruction loss, respectively. We implemented ℓ(·,·) as the mean squared error:

(35) $$\ell \left(\hat{y},y\right)={\left(\hat{y}-y\right)}^{2}$$

The predictive term directly optimises the fused representation for target-node regression. In contrast, the branch-specific reconstruction terms regularise the graph and tabular latent spaces to remain informative and complementary. Overall, the proposed architecture can be interpreted as a multi-view latent representation fusion model. The quantities $z_{g}$ and $z_{\mathrm{tab}}$ are learned latent representations obtained after non-linear transformations, branch-specific inductive biases, and projection stages. Their fusion enables the model to combine node-level relational reasoning with sample-level tabular abstraction in a unified prediction framework.
**Algorithm 2** Graph–Tabular Latent–Representation Fusion for Body Temperature Prediction**Require:** G=(V,E)|V={1,…,N}; ${\left\{x_{i}\right\}}_{i=1}^{N}$; ${\left\{c_{i}\right\}}_{i=1}^{N}$; ${\left\{o_{i}\right\}}_{i=1}^{N}$; $r\in R^{N}$; *t*; $y_{t}$; ${\left\{y_{i}\right\}}_{i\in \Omega }$; Ω⊆V; *L*; $\lambda_{f},\lambda_{g},\lambda_{\mathrm{tab}}$**Ensure:** ${\hat{y}}_{t}$; ${\hat{y}}^{\left(g\right)}$, ${\hat{y}}^{\left(\mathrm{tab}\right)}$; L   **Graph branch**  1:**for** i=1 to *N* **do**  2:       ${\tilde{x}}_{i}\leftarrow [x_{i}\;∥\;c_{i}\;∥\;o_{i}]$  3:       $h_{i}^{\left(0\right)}\leftarrow \mathrm{Drop}\left(\sigma_{\mathrm{act}}\left(\mathrm{BN}\left(W_{\mathrm{in}}{\tilde{x}}_{i}+b_{\mathrm{in}}\right)\right)\right)$  4:**end for**  5:**for** ℓ=1 to *L* **do**  6:       **for** i=1 to *N* **do**  7:             ${\hat{h}}_{i}^{\left(\ell \right)}\leftarrow M^{\left(\ell \right)}\left(\{h_{j}^{\left(\ell -1\right)}:j\in N\left(i\right)\cup \left\{i\right\}\},E\right)$  8:             $u_{i}^{\left(\ell \right)}\leftarrow \mathrm{Drop}\left(\sigma_{\mathrm{act}}\left(\mathrm{BN}\left({\hat{h}}_{i}^{\left(\ell \right)}\right)\right)\right)$  9:             $h_{i}^{\left(\ell \right)}\leftarrow h_{i}^{\left(\ell -1\right)}+u_{i}^{\left(\ell \right)}$10:       **end for**11:**end for**12:$h_{t}\leftarrow h_{t}^{\left(L\right)}$13:$O_{t}\leftarrow \left\{\;i\in V\;|\;i\neq t\;\mathrm{and}\;o_{i}=1\;\right\}$14:**if** $|O_{t}|>0$ **then**15:       ${\bar{h}}_{\mathrm{ctx}}\leftarrow \frac{1}{|O_{t}|}\sum_{i\in O_{t}}h_{i}^{\left(L\right)}$16:**else**17:       $V_{\mathrm{valid}}\leftarrow V$18:       ${\bar{h}}_{\mathrm{ctx}}\leftarrow \frac{1}{|V_{\mathrm{valid}}|}\sum_{i\in V_{\mathrm{valid}}}h_{i}^{\left(L\right)}$19:**end if**20:$z_{g}\leftarrow f_{g}\left([h_{t}\;∥\;{\bar{h}}_{\mathrm{ctx}}]\right)$21:${\hat{y}}^{\left(g\right)}\leftarrow d_{g}\left(z_{g}\right)$**Tabular branch**22:$I_{t}\leftarrow \left\{\;i\in V\;|\;i\neq t\;\right\}$23:$r_{I_{t}}\leftarrow {\left[\;r_{i}\;\right]}_{i\in I_{t}}$24:$z_{\mathrm{tab}}\leftarrow f_{\mathrm{tab}}\left(r_{I_{t}}\right)$25:${\hat{y}}^{\left(\mathrm{tab}\right)}\leftarrow d_{\mathrm{tab}}\left(z_{\mathrm{tab}}\right)$**Latent-level fusion and prediction**26:$z_{f}\leftarrow \left[z_{g}\;∥\;z_{\mathrm{tab}}\right]$27:${\hat{y}}_{t}\leftarrow f_{h}\left(z_{f}\right)$**Training objective**28:$L_{\mathrm{pred}}\leftarrow \ell \left({\hat{y}}_{t},y_{t}\right)$29:$L_{g}\leftarrow \frac{1}{\left|\Omega \right|}\sum_{i\in \Omega }\ell \left({\hat{y}}_{i}^{\left(g\right)},y_{i}\right)$30:$L_{\mathrm{tab}}\leftarrow \frac{1}{\left|\Omega \right|}\sum_{i\in \Omega }\ell \left({\hat{y}}_{i}^{\left(\mathrm{tab}\right)},y_{i}\right)$31:$L\leftarrow \lambda_{f}L_{\mathrm{pred}}+\lambda_{g}L_{g}+\lambda_{\mathrm{tab}}L_{\mathrm{tab}}$32:**return** ${\hat{y}}_{t}$, ${\hat{y}}^{\left(g\right)}$, ${\hat{y}}^{\left(\mathrm{tab}\right)}$, L

## 4. Experimental Setup

This section describes the experimental protocol used to evaluate body temperature prediction from thermal facial landmark data. The experiments were designed to assess model performance under complete, missing, and imputed landmark-temperature conditions. First, the TFD68 dataset was prepared as a landmark-temperature matrix. Next, structured and random missingness were simulated using MNAR and MCAR settings. Missing values were then reconstructed using Correlation-Based Multiple Imputation with Local *k*-Neighbour Matching (CMILK) to produce imputed datasets. Finally, graph-based, tabular, and multi-view fusion models were evaluated under a subject-wise cross-validation protocol.

### 4.1. Dataset Preparation

TFD68 was used for this experiment [4]. The dataset consists of frontal thermal facial images from 135 individuals, including 68 males and 67 females aged 18–52 years, primarily of Asian descent. Frontal images were selected because they provide the most complete visibility of facial landmarks, with most of the 68 landmarks remaining non-occluded. This makes them suitable for constructing the complete baseline landmark-temperature matrix before introducing controlled missingness. Using frontal samples also ensures that missingness in later experiments is introduced by the designed MNAR and MCAR protocols rather than by uncontrolled landmark absence in the original data.

**Figure 4:**
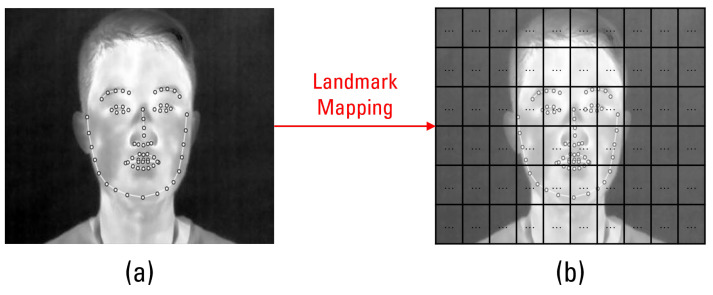
Facial landmark mapping used for thermal landmark temperature extraction. All 68 landmarks are mapped from (**a**) to (**b**) to extract the corresponding temperature value at each landmark location.

Each thermal image was manually annotated using the 68-point facial landmark scheme, as shown in Figure 4a. The corresponding temperature values for each landmark were extracted from the raw thermal data, as illustrated in Figure 4b. These extracted landmark temperatures were aggregated into a 135×68 matrix, where each row corresponds to a subject and each column to a facial landmark.

In this study, landmark 40 was selected as the target landmark for body temperature prediction. According to the adopted landmark mapping, this landmark corresponds to the inner canthus of the eye. The inner canthus is widely used in infrared thermography because it is reported to have a stronger and more stable correlation with core body temperature than many other facial regions, and is less sensitive to short-term ambient fluctuations [13,15,16]. While tympanic measurement remains a clinical gold standard [42], it is impractical for high-throughput, non-contact screening. Therefore, using the inner canthus temperature as the prediction target provides a practical compromise between physiological relevance and operational feasibility. Although this study focuses on the inner canthus due to its clinical relevance, the formulation is target-agnostic and can be extended to other landmarks or multi-target prediction.

All procedures were approved by the Heriot-Watt University School of Engineering and Physical Sciences Research Ethics Committee, and written informed consent was obtained from every participant before data collection.

### 4.2. Missing Dataset

#### 4.2.1. Missing Not at Random

To model occlusion-driven missingness, the MNAR datasets were generated using the 3D head-pose simulation protocol adopted by Ng. Y.C. et al. CMILK [2,3], and subsequently validated against real occlusion statistics from TFD68. The purpose of this design is to avoid arbitrary or uniformly random landmark removal. Instead, the missingness mechanism is made dependent on head pose, landmark location, and accessory type, which is consistent with an MNAR setting.

The empirical occlusion analysis was first performed using the real TFD68 annotations. For this analysis, the training, validation, and test splits were used, while the challenging split was excluded because the corresponding occlusion-rich image sets are incomplete. Mixed or uncertain accessory cases were also excluded to avoid ambiguous scenario definitions. After these exclusions, 123 participants were retained as the statistical reference for assessing real occlusion behaviour. Three occlusion scenarios were considered: self-occlusion, glasses occlusion, and face-mask occlusion. The self-occlusion images were available for all retained participants, while glasses and face-mask cases accounted for 55.28% and 44.72% of the retained accessory-occlusion cases, respectively.

For each retained TFD68 occlusion image, the 68 landmark visibility annotations were converted into an occlusion record indicating whether each landmark was visible or missing. The images were then grouped according to occlusion scenario and head pose. Within each group, we counted how frequently each landmark was annotated as missing across the retained participants. This frequency was used as the empirical missing probability of that landmark under the corresponding scenario. This analysis provides a data-driven reference distribution across the 68 landmarks and allows simulated MNAR masks to be compared with real TFD68 occlusion behaviour.

The simulated MNAR masks were generated using the CMILK-style 3D head-pose protocol. The 3D head model was rotated across yaw $\psi \in [-90^{\circ },90^{\circ }]$ and pitch $\theta \in [-30^{\circ },30^{\circ }]$ in $15^{\circ }$ increments, producing 65 head-pose configurations. For each pose, landmark self-occlusion was determined using a ray–mesh intersection test from a fixed virtual camera viewpoint. A landmark was marked as occluded when the line of sight from the camera to the landmark intersected the 3D head surface before reaching the landmark. This produces a physically interpretable pose-dependent visibility prior, where larger yaw and pitch angles naturally increase self-occlusion in anatomically plausible regions.

Three MNAR scenarios were then generated for each of the 65 poses. In the self-occlusion scenario, only the geometric visibility mask from the 3D head model was used. In the glasses scenario, the same self-occlusion mask was used as the base, and the eye-region landmarks were additionally marked as missing to represent occlusion by glasses. In the face-mask scenario, the self-occlusion mask was again used as the base, and the nose, mouth, and lower-jaw landmarks were additionally marked as missing to represent mask-related obstruction. Thus, each accessory condition was constructed on top of the same pose-dependent geometric self-occlusion process. The resulting simulated missingness covered a missingness range of approximately 5.88% to 86.76%, corresponding to about 6–87% of the 68 landmarks.

Table 1 compares the real TFD68 occlusion annotations with the simulated MNAR masks used in this study. Both settings contain 65 pose configurations per participant, 68 landmarks per image, and the same three occlusion scenarios: self-occlusion, glasses, and face mask. The real TFD68 annotations span a missingness range of 0.00% to 98.53%, while the simulated MNAR masks span a range of 5.88% to 86.76%. Although the simulation does not reproduce every extreme case observed in the real annotations, it covers a broad range of occlusion severity from mild to severe missingness. The mean number of missing landmarks is also comparable between TFD68 and the simulated MNAR masks, with 32.12 and 33.95 missing landmarks per image, respectively. Similarly, the median missing landmark count is 33.00 for TFD68 and 36.00 for the simulated MNAR setting. These results indicate that the simulated masks approximate the overall severity of missingness in real TFD68 occlusion cases while preserving a controlled, reproducible MNAR structure for evaluation.

After the MNAR masks were generated, they were applied to the direct frontal landmark-temperature profiles of the participants for yaw $0^{\circ }$ and pitch $0^{\circ }$. These frontal samples were used because they provide the most complete reference temperature profiles before artificial missingness is introduced. For the final MNAR dataset construction, the frontal landmark-temperature profiles of all available participants were used, including the challenging split, giving 135 individuals in total. The data were organised as a table, with each row representing one participant and each column representing one of the 68 landmark temperatures. For each MNAR condition, the generated mask determined which landmark-temperature entries were removed from this table.

The 65 pose configurations and three occlusion scenarios yielded 65×3=195 MNAR datasets. To prevent the fixed row order of participants from influencing the imputation or prediction outcome, the masks were applied using a circular rolling procedure across participants. In this procedure, the pose–scenario masks were shifted across participant rows, ensuring that each individual experienced MNAR missingness at least once and that no participant was always associated with the same mask position.

**Figure 5:**
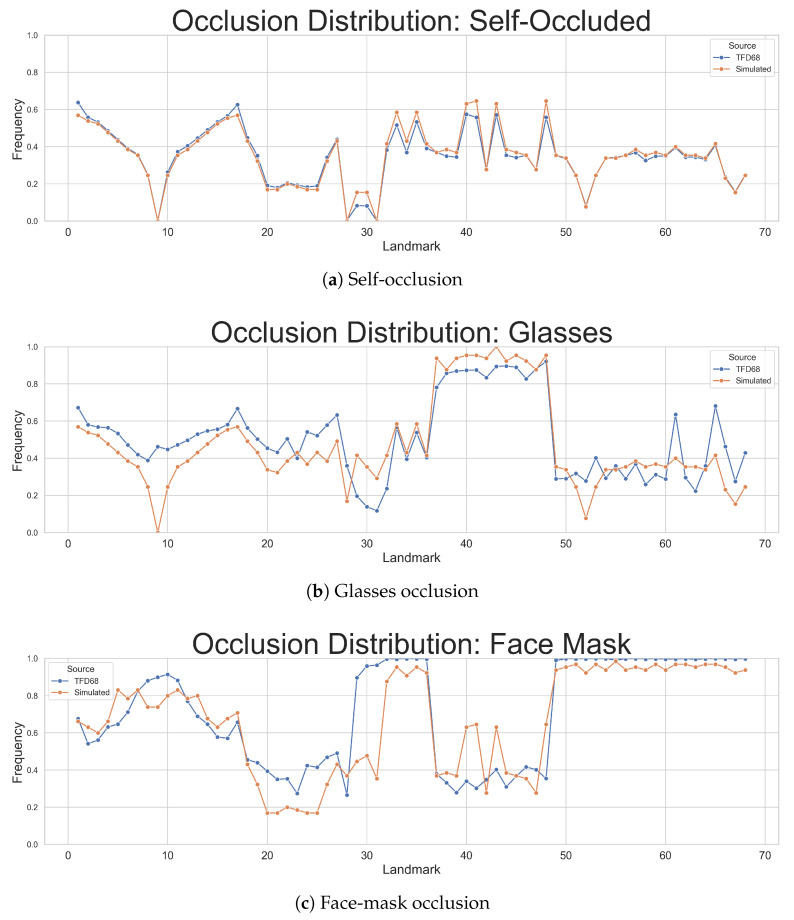
Landmark-wise occlusion probability distributions for real TFD68 annotations and simulated MNAR masks under the three occlusion scenarios. The plotted values show landmark-wise probabilities aggregated across the 65 pose configurations. The close correspondence between real and simulated curves indicates that the generated MNAR masks preserve the major landmark-level occlusion patterns observed in TFD68.

**Figure 6:**
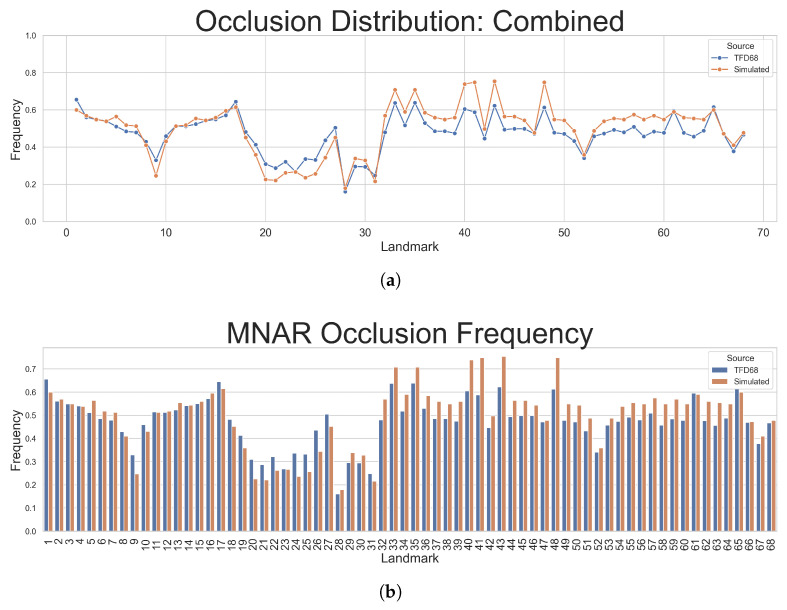
Overall landmark-wise agreement between real TFD68 occlusion annotations and simulated MNAR masks after combining all occlusion scenarios. The two subfigures present the same combined landmark-wise occlusion distribution in complementary formats. (**a**) The line plot compares the occlusion frequency of each of the 68 landmarks and highlights the overall trend across landmark indices. (**b**) The grouped bar plot provides a more detailed landmark-by-landmark comparison between the real annotations and the simulated masks. Any visual overlap between the real and simulated profiles reflects close agreement between the two distributions.

To verify the realism of the generated MNAR datasets, the simulated missingness distributions were compared against the real TFD68 occlusion annotations. First, the self-occlusion distribution was compared with the corresponding real TFD68 pose-induced occlusion distribution. Second, the glasses and face-mask simulations were compared with their respective real TFD68 accessory-occlusion distributions. Third, a combined landmark-wise distribution across all scenarios was compared against the combined real TFD68 occlusion distribution. These comparisons are shown in Figure 5 and Figure 6.

Figure 5 compares the landmark-wise occlusion probabilities between the real TFD68 annotations and the simulated MNAR masks for the three scenarios. In the self-occlusion case, the simulated curve closely matches the real TFD68 distribution, reflecting the pose-driven visibility structure of the 3D head model. In the glasses scenario, both real and simulated distributions show high occlusion probabilities around the periocular landmarks, particularly in the left- and right-eye regions. In the face-mask scenario, the highest occlusion probabilities occur around the nose, mouth, and lower-face landmarks, matching the expected anatomical effect of mask occlusion. These scenario-specific distributions show that the simulated MNAR masks reproduce the main landmark-wise patterns observed in real occlusion data, rather than behaving like random landmark removal.

Figure 6 further summarises the agreement between real and simulated occlusion patterns after combining the three scenarios. Both the line plot and grouped bar plot show that the simulated MNAR masks reproduce the overall landmark-wise trend of the real TFD68 occlusion annotations. The simulated distribution preserves the relative variation in missingness across the 68 landmarks, including higher missing frequencies around landmarks affected by pose-induced contour visibility, periocular obstruction, and lower-face occlusion. This confirms that the simulated masks do not assign uniform missing probabilities across landmarks, but instead follow structured landmark-dependent missingness.

Some differences between the real and simulated distributions are expected. The simulated masks are generated from controlled pose- and scenario-specific rules. In contrast, the real annotations also include subject-specific variation due to facial shape, accessory placement, head-pose execution, and annotation visibility. Nevertheless, the overall similarity between the real and simulated landmark-wise distributions supports the realism of the reconstructed MNAR masks. This indicates that the proposed MNAR construction is not based on arbitrary or uniformly random landmark removal, but preserves the dominant landmark-wise occlusion tendencies observed in TFD68 while retaining a reproducible simulation protocol.

The quantitative agreement metrics in Table 2 further confirm that the simulated MNAR masks are consistent with the real TFD68 occlusion distributions at the landmark level. For self-occlusion, the landmark-wise Pearson and Spearman correlations are 0.981 and 0.972, respectively, with an mean absolute error (MAE) of 0.020, indicating very close agreement between the real and simulated pose-driven occlusion patterns. For glasses occlusion, the Pearson and Spearman correlations are 0.852 and 0.797, respectively, with an MAE of 0.104, showing that the simulated masks capture the main periocular occlusion behaviour. For face-mask occlusion, the corresponding Pearson and Spearman correlations are 0.845 and 0.850, with an MAE of 0.107, confirming that the simulated masks preserve the lower-face occlusion behaviour observed in TFD68. Together, these visual and quantitative comparisons demonstrate that the MNAR simulation is a realistic, structured alternative to MCAR for evaluating occlusion-driven missingness.

#### 4.2.2. Missing Completely at Random

For the MCAR condition, nine independent dataset versions were generated with randomly missing landmarks at 10%, 20%, …, 90% overall missing rates, following Ng. Y.C. et al. CMILK [2,3]. For each target missing rate, landmark entries were selected uniformly at random across samples and landmarks. Unlike MNAR, MCAR does not model structured occlusion patterns; instead, it provides a baseline for evaluating performance under unstructured, noise-like missingness.

### 4.3. Imputed Dataset

Although graph-based methods can operate under partial missingness when observation indicators and masking strategies are used, their predictive performance is expected to degrade as the proportion of missing values increases. This is particularly relevant for landmark-temperature prediction, where high missingness can remove important neighbouring or correlated landmarks needed for reliable inference. Therefore, imputation is introduced as a preprocessing step to reconstruct missing landmark temperatures and enable graph-based, tabular, and multi-view models to exploit the full landmark-temperature structure better.

To evaluate a more realistic prediction scenario, imputed datasets were generated from the missing landmark-temperature datasets described in Section 4.2. In practical thermal facial analysis, landmark temperatures may be unavailable due to head pose, self-occlusion, glasses, face masks, sensor limitations, or annotation-related missingness. Rather than evaluating only on incomplete inputs, we also assess whether reconstructing the missing landmark temperatures improves downstream body temperature prediction.

Following the imputation protocol of Ng. Y.C. et al. [2,3], missing temperature values were imputed using CMILK. CMILK exploits inter-landmark thermal correlations to identify locally similar landmark-temperature patterns, while the genetic algorithm (GA) optimises the imputation hyperparameters. Specifically, the neighbourhood size *k* and convergence threshold ε were selected using GA-based optimisation before reconstructing the missing landmark values.

For each MNAR and MCAR missing dataset, CMILK was applied to estimate the missing landmark temperatures. This produced corresponding MNAR- and MCAR-imputed datasets, in which the originally missing entries were replaced with their imputed values, while the observed entries were retained. The resulting imputed datasets were saved and subsequently used as fixed inputs for downstream model evaluation. This separation ensures that all prediction models are evaluated on consistent imputed inputs.

The use of imputed datasets allows the study to compare three distinct evaluation conditions: complete data, missing data, and imputed data. The complete condition represents the ideal complete-input setting; the missing condition measures model robustness under incomplete landmark availability; and the imputed condition evaluates whether restoring missing landmark temperatures improves target-node or body temperature prediction. This design enables a direct assessment of the practical benefit of imputation for graph-based, tabular, and multi-view learning models.

### 4.4. Dataset Split and Cross-Validation

All experiments were conducted using a subject-wise data-splitting protocol to ensure strict separation of identities between the training and test sets. The full dataset was first divided into training, validation, and hold-out test subsets at the subject level. This means that all samples from a given subject were assigned to a single subset, preventing subject-level data leakage.

Cross-validation was performed only within the training split. Specifically, a five-fold subject-wise cross-validation protocol with $K_{\mathrm{cv}}=5$ was used to support model selection, hyperparameter tuning, and training stability assessment. In each cross-validation iteration, the training subjects were partitioned into five folds: one for validation and four for model fitting. The independent test split was not used during cross-validation and was kept fixed as a hold-out dataset for final evaluation.

The same hold-out test split was used across all evaluated methods and data conditions, including complete, MNAR-missing, MNAR-imputed, MCAR-missing, and MCAR-imputed datasets. This ensures that performance differences are attributable to the model or data condition rather than differences in test-subject composition. In particular, the missing and imputed datasets were evaluated using the same held-out subjects as the complete non-missing condition, enabling direct comparison between complete, missing, and imputed inputs under identical testing conditions.

### 4.5. Comparison Methods

To evaluate the proposed framework, we compared graph-based, tabular-based, and graph–tabular fusion models under the same experimental conditions. These comparison methods are direct instantiations of the generic formulation introduced in Section 3.2. Specifically, the graph-based methods correspond to different choices of the message-passing operator $M^{\left(\ell \right)}\left(\cdot \right)$ in Equation (11), while the tabular methods correspond to different choices of the tabular encoder $f_{\mathrm{tab}}\left(\cdot \right)$ in Equation (26). In the full fusion setting, the resulting graph latent representation $z_{g}$ from Equation (21) and tabular latent representation $z_{\mathrm{tab}}$ from Equation (26) are concatenated according to Equation (28) and passed to the prediction head $f_{h}\left(\cdot \right)$ in Equation (29). Therefore, the comparison is designed to evaluate how different graph encoders, tabular encoders, and their latent-level combinations affect target-node regression performance.

Unless otherwise stated, the default implementation follows the notation defined in the methodology. The graph hidden dimension is set to $D_{h}=64$, the graph latent dimension is set to $D_{g}=64$, and the tabular latent dimension is set to $D_{\mathrm{tab}}=64$. The tabular hidden dimension used internally by the tabular encoders is set to 128, the fusion hidden dimension is set to 64, the number of graph message-passing layers is set to L=3, and the dropout rate is set to p=0.3. Since each graph contains N=68 facial landmarks, the graph branch receives one node per landmark. Following Equation (8), each node input is formed by concatenating the landmark temperature value, two-dimensional coordinate information, and a binary observation indicator. In this implementation, $F_{x}=1$ and $F_{c}=2$, resulting in a four-dimensional augmented node vector ${\tilde{x}}_{i}\in R^{4}$. This input is projected into the shared hidden space using Equation (10) before being processed by the stacked message-passing layers in Equations (11)–(13).

The tabular branch follows the equations in Equations (24) and (25). Since the dataset contains N=68 landmarks and the target landmark t=40 is removed to prevent direct target leakage, the tabular encoder receives a 67-dimensional input vector $r_{I_{t}}\in R^{67}$. Each tabular backbone maps this vector into $z_{\mathrm{tab}}\in R^{D_{\mathrm{tab}}}$, as defined in Equation (26). The graph and tabular auxiliary decoders correspond to Equations (22) and (27), while the final training objective follows Equation (34).

#### 4.5.1. Graph-Branch Backbones

All graph backbones follow the shared graph-branch formulation described in Section 3.2. The main difference between backbones is the message-passing operator $M^{\left(\ell \right)}\left(\cdot \right)$ in Equation (11). Specifically, we evaluate GraphSAGE, GCNs, GAT, and GINs to compare mean neighbourhood aggregation, normalised graph convolution, attention-based aggregation, and expressive sum-based aggregation, respectively [6,7,30,31].

To ensure a fair comparison, all graph backbones use the same input dimensionality, hidden dimension, number of graph layers, dropout rate, residual update structure, graph projection head, and auxiliary decoder. Thus, performance differences mainly reflect the choice of graph message-passing operator rather than differences in model depth or latent dimensionality. The detailed architecture of each graph backbone is summarised in Table 3.

#### 4.5.2. Tabular-Branch Backbones

Since the dataset contains 68 landmarks and the target landmark is removed, each tabular encoder receives a 67-dimensional input vector $r_{I_{t}}$, as defined in Equation (25). Each backbone maps this input into a 64-dimensional tabular latent representation $z_{\mathrm{tab}}$, corresponding to Equation (26).

We evaluate MLPs, CNN1D, Linear-ReLU, ResNet-style MLP, TabNet-style, and FT-Transformer encoders to compare different forms of tabular feature learning. These include dense non-linear feature interaction, local sequential pattern extraction, shallow projection, residual tabular learning, attentive feature selection, and transformer-based contextual feature modelling [8,10,11]. To ensure a fair comparison, all tabular backbones produce the same latent output dimension and use the same auxiliary decoder structure. Thus, performance differences mainly reflect the tabular encoder design rather than differences in output dimensionality or reconstruction setup. The detailed architecture of each tabular backbone is summarised in Table 4.

#### 4.5.3. Fusion Head

In the full fusion model, the graph-side and tabular-side latent representations are concatenated into a 128-dimensional fused vector. This fused representation is passed through a shallow prediction head with structure 128→64→1, with ReLU and dropout between the two linear layers.

### 4.6. Baseline References

To contextualise the proposed graph–tabular fusion models, the baseline references are implemented as stand-alone predictors. Each baseline receives only a single modality and predicts the target scalar directly, without graph–tabular interaction, auxiliary reconstruction losses, or fusion-specific heads. This design provides a modality-isolated estimate of predictive performance, enabling any improvement observed in the fusion pipeline to be interpreted more clearly as a gain from combining complementary graph and tabular representations rather than from backbone choice alone. Such a protocol is consistent with the broader benchmarking literature, which emphasises the importance of strong and transparent unimodal baselines before introducing more elaborate tabular or graph architectures [8,9].

The neural tabular baselines include a shallow Linear-ReLU model, MLPs, a CNN1D, a ResNet-style MLP, and an FT-Transformer. ResNet-style MLPs and FT-Transformers have been identified as strong tabular deep learning references in controlled benchmarking settings [8]. TabNet is also included as an attentive tabular model that performs sequential feature selection [10]. The graph baselines span representative message-passing families, namely GCNs, GraphSAGE, GATs, and GINs.

This baseline set covers a broad range of inductive biases under the same prediction target and evaluation protocol: low-capacity linear models, modern tabular deep architectures, and canonical graph neural networks. Accordingly, the baseline references do not merely test whether the fusion model outperforms weak comparators; rather, they assess whether graph–tabular fusion improves upon strong stand-alone references from both the tabular and graph domains [8,9].

Table 5 summarises the stand-alone baseline models used as reference predictors. The table should be interpreted as an architectural specification rather than a performance table. The first column groups baselines by input modality: “Tabular” models operate on the landmark-temperature vector, and “Graph” models operate on graph-structured landmark inputs. The second column lists the model family, while the third column describes the main architectural components used for implementation, including projection layers, hidden dimensions, residual or attention mechanisms, dropout, and output heads. All baselines are trained under the same supervised regression protocol and predict the same scalar response, but they do not perform graph–tabular fusion. Therefore, the table enables the reader to identify which unimodal reference architectures are used to contextualise the performance of the proposed fusion models.

### 4.7. Evaluation Metrics

Model performance was evaluated using root mean square error (RMSE) and mean absolute error (MAE). These metrics were selected because the task is formulated as a scalar regression problem: predicting the target landmark temperature ${\hat{y}}_{t}$ from the remaining landmark information. For an evaluation set containing *M* test samples, RMSE and MAE are defined as

(36) $$\mathrm{RMSE}=\sqrt{\frac{1}{M}\sum_{m=1}^{M}{\left({\hat{y}}_{t,m}-y_{t,m}\right)}^{2}}$$

and

(37) $$\mathrm{MAE}=\frac{1}{M}\sum_{m=1}^{M}\left|{\hat{y}}_{t,m}-y_{t,m}\right|$$

where $y_{t,m}$ and ${\hat{y}}_{t,m}$ denote the ground-truth and predicted target values for test sample *m*, respectively.

The same metrics were used for all evaluation conditions, including the complete dataset, MNAR-missing dataset, MNAR-imputed dataset, MCAR-missing dataset, and MCAR-imputed dataset. This ensures that model performance can be compared consistently across complete, incomplete, and reconstructed landmark-temperature inputs.

During testing, each trained method was evaluated on the held-out test set using an ensemble of the models obtained from the five cross-validation folds. For each evaluation set, each fold-specific model produced a prediction for each held-out test sample. These predictions were then aggregated across folds using the mean reducer:

(38) $${\hat{y}}_{t,m}=\frac{1}{K_{\mathrm{cv}}}\sum_{k=1}^{K_{\mathrm{cv}}}{\hat{y}}_{t,m}^{\left(k\right)}$$

where ${\hat{y}}_{t,m}^{\left(k\right)}$ denotes the prediction produced by the model trained in fold *k*, and $K_{\mathrm{cv}}=5$ is the number of cross-validation folds. The resulting ensemble prediction was compared against the ground-truth target value from the held-out test graph. This reference target remained the target value even when the input graph corresponded to a missing-data or imputed-data variant.

For each evaluation set, RMSE and MAE were computed over all held-out test samples using Equations (36) and (37). The resulting per-set metrics were then grouped by evaluation category. Let $q_{s}$ denote a per-set metric value, such as RMSE or MAE, for dataset variant *s*, and let $S_{c}$ denote the set of dataset variants belonging to category *c*, such as complete, MNAR-missing, MNAR-imputed, MCAR-missing, or MCAR-imputed. The category-level mean is computed as

(39) $${\bar{q}}_{c}=\frac{1}{|S_{c}|}\sum_{s\in S_{c}}q_{s}$$

and the corresponding standard deviation is computed as

(40) $$\sigma_{q,c}=\sqrt{\frac{1}{|S_{c}|-1}\sum_{s\in S_{c}}{\left(q_{s}-{\bar{q}}_{c}\right)}^{2}}$$

Thus, the reported standard deviations quantify the variability of per-set RMSE or MAE values within each evaluation category.

To assess whether latent-level fusion provides a statistically significant improvement over its single-branch counterparts, hypothesis testing was performed between each fusion model and its corresponding graph-only and tabular-only ablations. For a given metric, let $q_{s}^{\left(f\right)}$ denote the score of the fusion model on evaluation set *s*, and let $q_{s}^{\left(b\right)}$ denote the score of the corresponding baseline branch model, where *b* represents either the graph-only or tabular-only model. Since lower RMSE and MAE indicate better performance, the paired improvement is defined as

(41) $$d_{s}=q_{s}^{\left(b\right)}-q_{s}^{\left(f\right)}$$

A positive value of $d_{s}$ indicates that the fusion model achieved a lower error than the corresponding ablation model.

Paired *t*-tests were used to test whether the mean paired improvement was significantly greater than zero:

(42) $$t=\frac{\bar{d}}{s_{d}/\sqrt{|S_{c}|}}$$

where $\bar{d}$ and $s_{d}$ denote the mean and standard deviation of the paired improvements across the matched evaluation sets, respectively. In addition, the Wilcoxon signed-rank test was used as a non-parametric alternative to assess whether the fusion model consistently improved over the ablation model without assuming normality of the paired differences.

The effect size was reported using Cohen’s $d_{z}$ for paired samples:

(43) $$d_{z}=\frac{\bar{d}}{s_{d}}$$

Together, the paired *t*-test, Wilcoxon signed-rank test, and Cohen’s $d_{z}$ provide complementary evidence on whether the proposed fusion models offer statistically and practically meaningful improvements over their graph-only and tabular-only ablation counterparts.

### 4.8. Hardware and Software Environment

All experiments were conducted on a workstation equipped with an NVIDIA RTX 3060 GPU with 12 GB of VRAM, 32 GB RAM, and an Intel Core i7-9700 CPU. The operating system was Windows 11. The implementation was developed using Python 3.8.2 and PyTorch 2.4.1, with support for CUDA 12.4.

For efficiency evaluation, all models were evaluated using the same hardware and software environment. Inference time was measured with the trained model in evaluation mode and gradient computation disabled. The batch size was fixed at 16 for both training and inference, unless otherwise stated. FPS was computed from the measured inference time as the number of evaluated samples divided by the total inference duration. This setup ensures that training time, inference time, and FPS comparisons are measured under a consistent implementation environment.

### 4.9. Implementation Details and Hyperparameters

All models were trained under the same supervised regression protocol to ensure a fair comparison between stand-alone tabular baselines, stand-alone graph baselines, and graph–tabular fusion models. For each training sample, the target response was used only as the regression label. To prevent direct target leakage, the corresponding target entry in the input representation was replaced by a fixed sentinel value of −1 before being passed to the model. This operation was applied consistently across the tabular, graph, and fused settings.

For the tabular branch, the landmark-temperature vector was represented as a compact structured input. Unless otherwise stated, tabular models used a hidden dimension of 128, a latent output dimension of 64, ReLU activation, and dropout with probability p=0.3. The MLP backbone used two linear layers, (67→128→64), while the CNN1D backbone used two one-dimensional convolutional layers followed by adaptive average pooling and a 64-dimensional projection. The ResNet-style MLP used three residual blocks with expansion factor 2, and the FT-Transformer used 128-dimensional feature tokens, two Transformer encoder layers, eight attention heads, and a feed-forward dimension of 256. The TabNet-style model used four decision steps, hidden width 128, and relaxation parameter γ=1.3.

For the graph branch, each landmark was represented as a node, and the node feature vector was formed by concatenating the landmark temperature feature, spatial coordinate vector, and observation indicator. The graph models used an initial projection to a 64-dimensional hidden space followed by three residual message-passing layers. GCNs, GraphSAGE, GATs, and GINs were implemented using the same hidden width to ensure architectural comparability. GraphSAGE used mean aggregation, GATs used four attention heads, and GINs used internal MLP blocks with dimensions (64→64→64). Dropout with probability p=0.3 was applied where applicable.

For the fused models, the graph and tabular branches first encoded their respective views independently into 64-dimensional latent representations. These two latent vectors were concatenated to form a 128-dimensional fused representation, which was then passed to a fully connected prediction head with dimensions (128→64→1). ReLU activation was used between hidden layers, and the final layer produced a single scalar prediction.

All models were trained for up to 100 epochs with a batch size of 16. The Adam optimiser was used with a learning rate of 0.005 and weight decay of 0.0001. A CosineAnnealingLR scheduler was applied during training, and early stopping was used with a patience of 10 epochs based on validation performance. For fold-level prediction aggregation, the ensemble reducer was set to the mean.

The main supervised prediction loss was mean squared error (MSE). For the fused framework, auxiliary reconstruction losses were also used to regularise the graph and tabular latent representations, as defined in Equations (32) and (33). The reconstruction terms used Smooth L1 loss with β=0.1. The total training objective followed Equation (34), where the fused prediction loss weight, graph reconstruction loss weight, and tabular reconstruction loss weight were all set to 1.0.

Training, validation, and test splits were kept fixed across all baseline and fused models. The same missingness setting, imputation protocol, batch size, optimiser, scheduler, loss configuration, and evaluation metrics were applied across models to ensure that performance differences were attributable to the model architecture rather than to changes in data processing or training procedure.

## 5. Results and Discussion

### 5.1. Fusion and Baseline Results

Table 6 reports the predictive performance of the stand-alone baseline references under complete, missing, and imputed conditions. The raw missing-data setting shows a clear separation between graph and non-graph baselines. Under MNAR-missing inputs, tabular-only models such as Linear-ReLU, MLPs, CNN, ResNet-MLP, TabNet, and FT-Transformer degrade substantially, with $\bar{\mathrm{RMSE}}$ values above 31. This is expected because these models receive fixed-length tabular vectors and do not explicitly model observation status or relational structure when landmark values are absent. In contrast, graph baselines are more robust to raw missingness, with GATs achieving the lowest MNAR-missing $\bar{\mathrm{RMSE}}$ (1.356) and $\bar{\mathrm{MAE}}$ (1.119), followed by GCNs. This suggests that graph message passing and node-level observation information provide useful robustness when landmark availability is incomplete.

After CMILK-based imputation, the errors of all baselines decrease substantially under the MNAR-imputed condition, which most closely reflects realistic occlusion-driven missingness. The strongest stand-alone $\bar{\mathrm{RMSE}}$ is obtained by GINs (0.310), followed by GraphSAGE (0.315), GATs (0.317), and Linear-ReLU (0.317). For $\bar{\mathrm{MAE}}$, Linear-ReLU gives the lowest value (0.254), followed closely by MLPs (0.255), GINs (0.256), and TabNet (0.256). These results indicate that imputation restores the usefulness of compact tabular representations, while graph models remain competitive because they exploit landmark dependency structure.

Table 7 summarises the computational cost of the stand-alone baselines. The three fastest methods in total training time are Linear-ReLU (97.828 s), MLPs (116.281 s), and CNN (149.694 s), while the slowest are GATs (1627.387 s), GCNs (1243.198 s), and GINs (1042.571 s). This reflects the additional computational cost of graph message passing compared with direct tabular encoding.

For inference, the lightweight tabular models provide the highest throughput. MLPs give the fastest complete-data inference, while Linear-ReLU gives the fastest MNAR-missing, MCAR-missing, MNAR-imputed, and MCAR-imputed inference. In contrast, graph baselines have lower FPS because each prediction requires message passing over the landmark graph. Therefore, the graph baselines provide stronger robustness under raw missingness, but this robustness comes with a higher inference-time cost than lightweight tabular baselines.

Table 8 evaluates the proposed graph–tabular latent-representation fusion framework across different backbone combinations. Under MNAR-imputed evaluation, the top three $\bar{\mathrm{RMSE}}$ configurations are GATs + MLPs (0.296), GraphSAGE + MLPs (0.297), and GINs + Linear-ReLU (0.298). Their corresponding $\bar{\mathrm{MAE}}$ values are also highly competitive: GraphSAGE + MLPs achieves the lowest MNAR-imputed $\bar{\mathrm{MAE}}$ (0.243), followed closely by GINs + Linear-ReLU (0.244) and GATs + MLPs (0.245). These results show that the proposed fusion framework improves over the best stand-alone MNAR-imputed $\bar{\mathrm{RMSE}}$ of GINs (0.310), indicating that combining graph-side relational representations with tabular-side global feature interactions provides a more informative latent representation than either modality alone.

**Figure 7:**
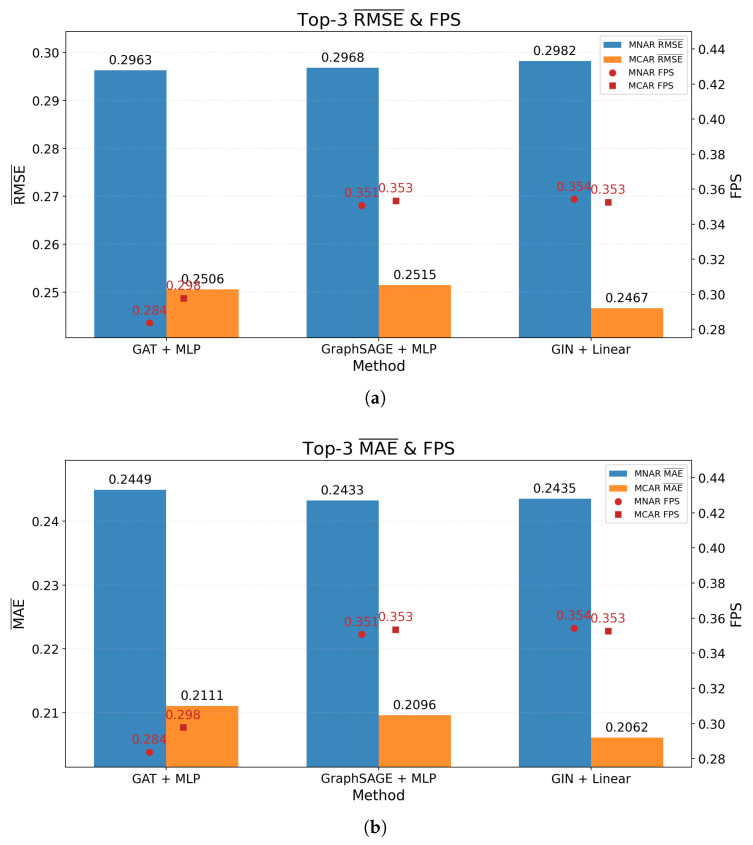
Accuracy–efficiency comparison of the top-three fused graph–tabular configurations under imputed missingness. Each subfigure compares prediction error and inference throughput for the same three fused methods. The blue and orange bars represent MNAR- and MCAR-imputed prediction errors, respectively, where lower values indicate better prediction accuracy. The red circular markers indicate MNAR inference throughput in FPS, while the red square markers indicate MCAR inference throughput in FPS, where higher values indicate faster inference. (**a**) $\bar{\mathrm{RMSE}}$–FPS comparison of the top-three fused methods. GAT + MLP achieves the lowest MNAR-imputed $\bar{\mathrm{RMSE}}$ among the selected configurations. (**b**) $\bar{\mathrm{MAE}}$–FPS comparison of the top-three fused methods. GraphSAGE + MLP achieves the lowest MNAR-imputed $\bar{\mathrm{MAE}}$, while GIN + Linear-ReLU provides the highest FPS and the lowest MCAR-imputed error among the selected configurations.

Figure 7 summarises the accuracy–efficiency trade-off of the three strongest fused graph–tabular configurations. The figure should be interpreted by comparing the prediction-error bars with the inference-throughput markers. In each subfigure, the blue and orange bars show the prediction errors obtained under MNAR- and MCAR-imputed conditions, respectively. The red circular markers indicate MNAR inference throughput, while the red square markers indicate MCAR inference throughput. Lower bars indicate better prediction accuracy, whereas higher markers indicate faster inference.

Figure 7a compares the top-three fused configurations using $\bar{\mathrm{RMSE}}$, while Figure 7b compares the same configurations using $\bar{\mathrm{MAE}}$. GATs + MLPs provides the lowest MNAR-imputed $\bar{\mathrm{RMSE}}$, GraphSAGE + MLPs provides the lowest MNAR-imputed $\bar{\mathrm{MAE}}$, and GINs + Linear-ReLU achieves the strongest MCAR-imputed accuracy. In terms of throughput, GINs + Linear-ReLU and GraphSAGE + MLPs are slightly faster than GATs + MLPs under the imputed settings. Therefore, the top fused configurations offer different accuracy–efficiency trade-offs: GATs + MLPs is most suitable when minimising $\bar{\mathrm{RMSE}}$ is prioritised, while GraphSAGE + MLPs and GINs + Linear-ReLU provide more favourable $\bar{\mathrm{MAE}}$/FPS balance.

The broader fused results further show that the choice of tabular branch strongly affects performance. MLP and Linear-ReLU pairings generally perform well under imputed conditions, whereas TabNet-based fusion tends to underperform, with GATs + TabNet, GINs + TabNet, GCNs + TabNet, and GraphSAGE + TabNet among the weaker MNAR-imputed configurations. This suggests that sequential attentive feature selection is not necessarily advantageous for this compact, continuous landmark-temperature vector. In contrast, simpler dense or linear tabular encoders appear to provide more stable complementary information when fused with graph embeddings.

Table 9 reports the training time, inference time, and FPS of the fused models. The three fastest training configurations are GINs + CNN1D (926.955 s), GINs + MLPs (978.554 s), and GCNs + CNN1D (1048.890 s). GAT-based configurations generally require longer training times due to the additional cost of attention-based neighbourhood weighting. The best MNAR-imputed $\bar{\mathrm{RMSE}}$ configuration, GATs + MLPs, requires 1459.675 s for training, which is higher than the fastest configurations but remains acceptable because training is performed offline.

For deployment, inference throughput is more relevant. With the updated timing results, fused models achieve lower FPS than lightweight tabular baselines, but they provide improved prediction accuracy when handling missing values. Under MNAR-imputed inference, the fastest fused configurations are GraphSAGE + Linear-ReLU (0.358 FPS), GINs + Linear-ReLU (0.354 FPS), and GraphSAGE + MLPs (0.351 FPS). Among the top-three accuracy configurations shown in Figure 7a,b, GINs + Linear-ReLU gives the highest throughput, GraphSAGE + MLPs provides a similar throughput with the lowest MNAR-imputed $\bar{\mathrm{MAE}}$, and GATs + MLPs prioritises $\bar{\mathrm{RMSE}}$ accuracy over speed. Thus, the fused models should be interpreted as accuracy-oriented configurations that remain computationally feasible, rather than as faster alternatives to lightweight stand-alone baselines.

**Figure 8:**
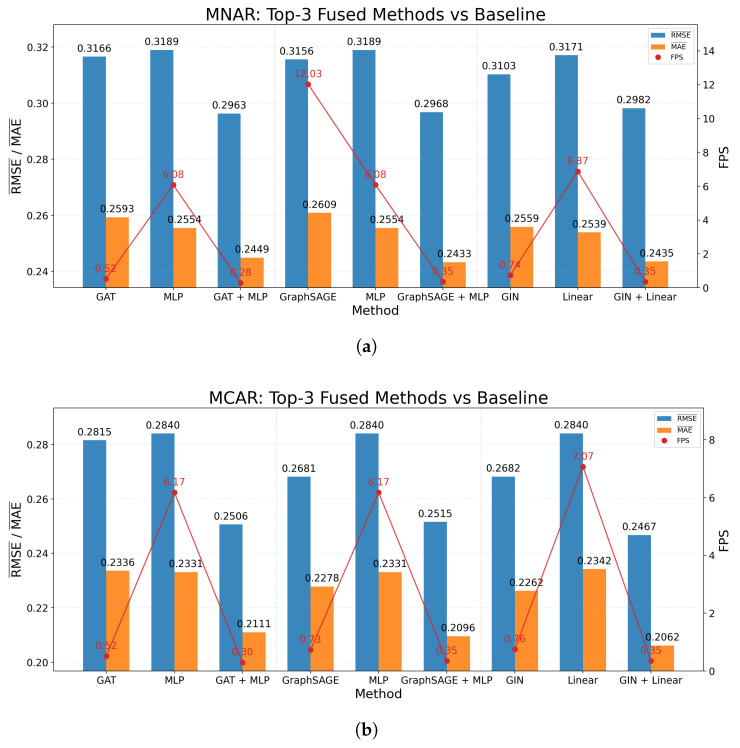
Comparison of the top-three fused methods against their corresponding stand-alone baselines under imputed missingness. Each group of three methods should be read as a graph-only baseline, a tabular-only baseline, and their fused graph–tabular model. The bars report prediction error, where lower $\bar{\mathrm{RMSE}}$ and $\bar{\mathrm{MAE}}$ indicate better accuracy, while the red markers report inference throughput in FPS, where higher values indicate faster inference. (**a**) MNAR-imputed comparison between the top-three fused methods and their corresponding stand-alone graph and tabular baselines. (**b**) MCAR-imputed comparison between the top-three fused methods and their corresponding stand-alone graph and tabular baselines. Overall, the fused models generally reduce prediction error relative to their individual graph-only and tabular-only counterparts, illustrating the benefit of latent-representation fusion under imputed missingness.

Figure 8 compares each top fused configuration with its corresponding stand-alone graph and tabular baselines. The figure should be interpreted in groups of three models: the graph-only baseline, the tabular-only baseline, and the fused graph–tabular model. For example, the GAT, MLP, and GAT + MLP group shows whether the fused model improves over both of its individual branches. In each group, lower $\bar{\mathrm{RMSE}}$ and $\bar{\mathrm{MAE}}$ bars indicate better prediction accuracy, while the red FPS marker indicates inference throughput. Figure 8a reports the comparison under MNAR-imputed evaluation, and Figure 8b reports the same comparison under MCAR-imputed evaluation.

Under MNAR-imputed evaluation, all three fused models reduce both $\bar{\mathrm{RMSE}}$ and $\bar{\mathrm{MAE}}$ relative to their individual baselines. GATs + MLPs reduces $\bar{\mathrm{RMSE}}$ by 6.41% relative to GATs and 7.09% relative to MLPs, while reducing $\bar{\mathrm{MAE}}$ by 5.55% and 4.11%, respectively. GraphSAGE + MLPs similarly improves over GraphSAGE by 5.96% in $\bar{\mathrm{RMSE}}$ and 6.75% in $\bar{\mathrm{MAE}}$, and over MLPs by 6.93% in $\bar{\mathrm{RMSE}}$ and 4.74% in $\bar{\mathrm{MAE}}$. GINs + Linear-ReLU gives smaller but still consistent MNAR improvements, reducing $\bar{\mathrm{RMSE}}$ by 3.90% relative to GINs and 5.96% relative to Linear-ReLU, while reducing $\bar{\mathrm{MAE}}$ by 4.85% and 4.10%, respectively. These results show that the fused models do not simply reproduce the performance of either branch, but combine complementary graph-side and tabular-side information to obtain lower prediction error.

**Figure 9:**
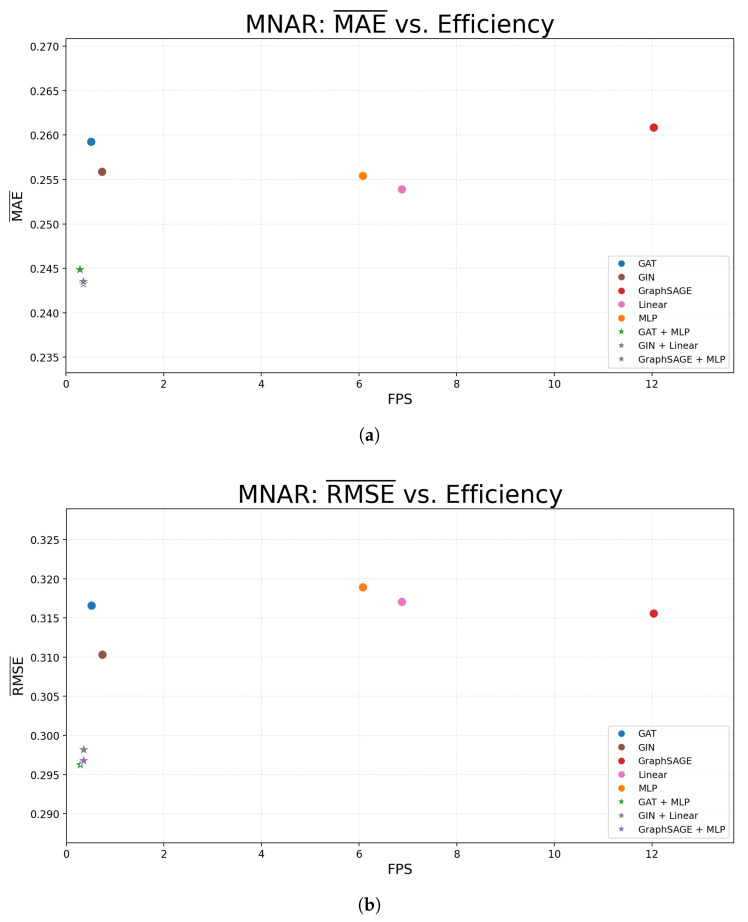
Accuracy–efficiency scatter plots for the top fused configurations and their corresponding stand-alone baselines under the MNAR-imputed condition. Circular markers represent stand-alone graph or tabular baselines, while star markers represent fused graph–tabular models. The horizontal axis reports inference throughput in FPS, and the vertical axis reports prediction error; therefore, methods closer to the lower-right region achieve lower error and higher throughput. (**a**) MNAR-imputed $\bar{\mathrm{MAE}}$ versus FPS. (**b**) MNAR-imputed $\bar{\mathrm{RMSE}}$ versus FPS. Overall, the fused models generally reduce prediction error, but do not necessarily increase FPS, indicating that latent-representation fusion improves accuracy while introducing an inference-speed trade-off.

To further visualise the relationship between prediction accuracy and deployment efficiency, Figure 9 compares the top fused configurations with their corresponding stand-alone graph and tabular baselines under the MNAR-imputed condition. The horizontal axis reports inference throughput in FPS, while the vertical axis reports prediction error. Therefore, methods located toward the lower-right region are preferable because they combine lower error with higher inference speed. Circular markers denote stand-alone baselines, while star markers denote fused graph–tabular models.

The scatter plots show that the fused configurations occupy a lower-error region than their individual baselines, but not necessarily a higher-throughput region. In the $\bar{\mathrm{MAE}}$ plot, GraphSAGE + MLPs achieves the lowest error among the compared models, while GINs + Linear-ReLU provides a similar $\bar{\mathrm{MAE}}$ with slightly higher FPS among the fused configurations. In the $\bar{\mathrm{RMSE}}$ plot, GATs + MLPs achieves the lowest error, while GINs + Linear-ReLU remains the fastest among the top fused models. These trends complement the bar-chart comparisons in Figure 8: the fused models reduce prediction error relative to their graph-only and tabular-only counterparts, but this improvement comes with an inference-speed trade-off compared with lightweight tabular baselines.

The improvement is even more pronounced under the MCAR-imputed evaluation. GATs + MLPs reduces $\bar{\mathrm{RMSE}}$ by 10.98% relative to GATs and 11.76% relative to MLPs, while reducing $\bar{\mathrm{MAE}}$ by 9.63% and 9.44%, respectively. GraphSAGE + MLPs reduces $\bar{\mathrm{RMSE}}$ by 6.19% relative to GraphSAGE and 11.44% relative to MLPs, with $\bar{\mathrm{MAE}}$ reductions of 7.99% and 10.08%. GINs + Linear-ReLU provides the strongest MCAR improvement among the three, reducing $\bar{\mathrm{RMSE}}$ by 8.02% relative to GINs and 13.13% relative to Linear-ReLU, while reducing $\bar{\mathrm{MAE}}$ by 8.84% and 11.96%, respectively. This suggests that the fused representation is particularly effective when imputation restores a more complete feature structure after random missingness.

**Figure 10:**
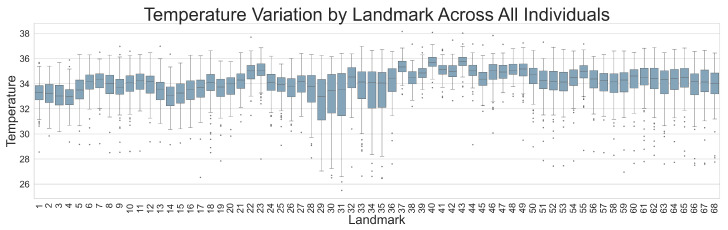
Landmark-wise temperature variation across all individuals. Each boxplot summarises the distribution of measured temperatures for one facial landmark, showing both spatial variation across landmark locations and inter-subject variation within each landmark. The box indicates the interquartile range, the central line indicates the median, and the whiskers show the non-outlier range. Dots represent individual outlier measurements, corresponding to landmark-temperature values that fall outside the whisker range for that landmark.

Figure 10 further contextualises the prediction errors by showing the landmark-wise temperature distribution across all individuals. The distributions reveal substantial spatial and inter-subject variability across the 68 facial landmarks, with an average intra-face landmark-temperature variation of approximately 5.285 °C. This confirms that facial temperature is highly heterogeneous rather than uniformly distributed. Consequently, single-region measurement strategies may be sensitive to landmark choice, anatomical location, local perfusion, environmental effects, and subject-specific thermal patterns. This observation supports the need for multi-landmark modelling, where information from multiple facial regions is jointly considered rather than relying on a single fixed region.

Despite this variability, the best MNAR-imputed fused configurations achieve $\bar{\mathrm{MAE}}$ values of approximately 0.243–0.245 °C. This error magnitude is small relative to the observed landmark-temperature variation, indicating that the proposed graph–tabular latent-representation fusion model can learn a compact representation that normalises complex spatial and inter-subject thermal differences. In other words, although the raw facial thermal profile exhibits large variation across landmarks and individuals, the learned fusion representation reduces this variability to a substantially lower prediction error.

Beyond statistical significance, the practical value of the proposed fusion models should, therefore, be interpreted in terms of both temperature-estimation error and deployment throughput. Non-contact temperature-measurement standards and evaluation studies commonly discuss acceptable laboratory accuracy within several tenths of a degree Celsius. In particular, IEC 80601-2-59 [43] defines requirements for screening thermographs used for human febrile temperature screening. At the same time, non-contact infrared clinical thermometer standards such as ASTM E1965 [44] and ISO 80601-2-56 [45] specify requirements for infrared or clinical thermometers used for patient or body-temperature measurement. These standards provide a practical reference for interpreting prediction errors within the range of several tenths of a degree Celsius, although they are defined for measurement devices rather than predictive models. Screening thermograph evaluations also commonly discuss accuracy around ±0.5 °C under controlled conditions [46,47,48]. Although these standards are defined for clinical measurement devices rather than predictive models, they provide a useful practical reference for interpreting the magnitude of the prediction error. In this context, an $\bar{\mathrm{MAE}}$ of approximately 0.243–0.245 °C indicates an average absolute prediction error of about one-quarter of a degree Celsius, although it should not be interpreted as a strict bound for every individual prediction.

The relative improvement of approximately 4–7% over the corresponding stand-alone baselines, therefore, represents a measurable reduction in model-induced error within a practically relevant temperature range. When considered together with inference throughput, different fused models offer different deployment trade-offs: GATs + MLPs provides the lowest MNAR-imputed $\bar{\mathrm{RMSE}}$, GraphSAGE + MLPs provides the lowest MNAR-imputed $\bar{\mathrm{MAE}}$, and GINs + Linear-ReLU offers the highest FPS among the top configurations. Thus, the fused models improve robustness under occlusion-driven missingness and heterogeneous landmark-temperature distributions, while the reported FPS values quantify the additional computational cost required to obtain this improvement. These results should be interpreted as improving the robustness of non-contact screening-oriented prediction rather than replacing calibrated clinical thermometry, which still requires appropriate calibration, environmental control, and subject acclimatisation [46].

The fused models do not show a universal runtime advantage over stand-alone baselines. Their inference cost is higher than that of lightweight tabular models because the fused pipeline combines graph and tabular encodings before latent-level prediction. Therefore, FPS results should be interpreted as evidence of computational feasibility rather than as a measure of speed superiority. The main advantage of the fused framework is its lower prediction error under imputed missingness, while the runtime results quantify the deployment cost associated with this improvement.

### 5.2. Statistical Analysis

**Figure 11:**
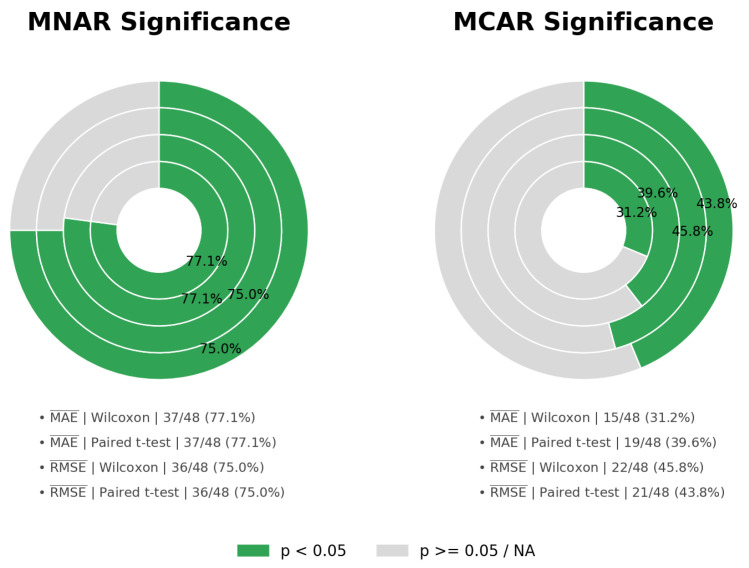
Share of statistically significant fused–baseline analysis pairs under the MNAR-imputed condition. Green segments indicate the percentage of comparisons with p<0.05, while grey segments indicate non-significant or unavailable comparisons. The results summarise paired *t*-test and Wilcoxon signed-rank outcomes for $\bar{\mathrm{RMSE}}$ and $\bar{\mathrm{MAE}}$ across all fused-method versus baseline comparisons.

To more comprehensively assess the reliability of the proposed fusion framework, paired statistical testing was conducted for all fused-model versus baseline comparisons under the MNAR-imputed condition. Specifically, each fused configuration was compared against its corresponding graph-only and tabular-only baseline using paired *t*-tests and Wilcoxon signed-rank tests for both $\bar{\mathrm{RMSE}}$ and $\bar{\mathrm{MAE}}$. Since the complete set of pairwise results is too large to present clearly in a single table, Figure 11 summarises the overall outcome by showing the proportion of fused–baseline analysis pairs that were statistically significant at p<0.05. For completeness and interpretability, Table 10 then reports representative detailed results for three strong fused configurations: GATs + MLPs, GraphSAGE + MLPs, and GINs + Linear-ReLU.

The summary in Figure 11 shows that the fusion framework produces statistically reliable improvements more consistently under MNAR-imputed evaluation than under MCAR-imputed evaluation. For the MNAR-imputed condition, significant improvements are observed in 37/48 comparisons for $\bar{\mathrm{MAE}}$ using both the Wilcoxon signed-rank test and paired *t*-test, corresponding to 77.1%. For $\bar{\mathrm{RMSE}}$, 36/48 comparisons are significant for both tests, corresponding to 75.0%. This indicates that, under realistic occlusion-driven missingness, the proposed fusion framework usually improves prediction error in a statistically reliable manner.

Under MCAR-imputed evaluation, the proportion of significant improvements is lower, ranging from 31.2% to 45.8% depending on the metric and test. This does not contradict the numerical improvements observed for the strongest fused configurations in Figure 8; rather, it shows that the benefit of fusion is more consistent under structured MNAR missingness than under random missingness. Since MCAR removes entries independently of landmark location, imputation can restore a more regular feature structure, allowing some stand-alone baselines to remain competitive. In contrast, MNAR missingness is pose- and occlusion-dependent, making the complementary graph and tabular representations more beneficial.

At the same time, approximately 28–32% of the tested pairs are not statistically significant. This is an important and expected outcome rather than a contradiction of the framework’s effectiveness. First, the proposed method is a fusion framework, not a single fixed architecture. Its benefit, therefore, depends on whether the selected graph encoder and tabular backbone provide complementary information. Prior graph–tabular and graph–descriptor fusion studies show that fusion is most effective when the two branches capture different aspects of the data, such as relational structure and non-graph descriptors, rather than redundant representations [33,34,35,36]. Therefore, some backbone pairings are expected to provide strong complementarity, while others may contribute overlapping or weakly informative features.

Second, several stand-alone baselines are already competitive after CMILK-based imputation, leaving only a limited margin for further improvement. This is consistent with tabular-learning studies showing that simple or well-tuned tabular models can remain strong baselines and that no single specialised architecture is uniformly superior across all structured datasets [8,9]. In such cases, a fused model may still reduce the mean error, but the improvement may be too small or too inconsistent across paired evaluation sets to reach statistical significance. Third, statistical significance depends not only on the average improvement but also on its consistency. Some fused configurations show larger variability across MNAR-imputed sets, which weakens the paired evidence even when the mean performance is slightly better. Finally, weaker pairings, particularly those involving tabular branches that are less suited to compact continuous landmark-temperature vectors, may not consistently enhance the graph representation. This explains why a non-trivial minority of fused–baseline comparisons are non-significant, while the overall majority of significant comparisons still supports the usefulness of graph–tabular latent-representation fusion.

Overall, the statistical analysis reinforces two key conclusions. First, the fusion strategy is broadly effective, as reflected by the clear majority of significant fused–baseline comparisons. Second, the presence of a non-trivial minority of non-significant comparisons shows that fusion is not automatically beneficial for every backbone pairing. This is precisely why the framework should be interpreted as a flexible design space rather than a single universally optimal architecture. In practice, configurations such as GATs + MLPs, GraphSAGE + MLPs, and GINs + Linear-ReLU emerge as particularly strong choices because they combine favourable predictive accuracy with statistically supported improvements over their stand-alone counterparts.

### 5.3. Graph Construction Sensitivity Analysis

To further validate the graph-construction design, two control experiments were conducted after the main baseline and fusion evaluations. The first experiment evaluates the sensitivity of the PCC threshold τ, while the second compares graph topologies constructed using Pearson, Spearman, and Kendall correlation coefficients. Since graph construction directly affects only the graph topology, the analysis is performed using a graph-only setting to isolate the effect of the edge definition without introducing additional variation from the tabular branch or fusion head. Following the fusion results in Section 5.1, GATs is used as the graph evaluator because the GAT-based fused configuration achieved the strongest MNAR-imputed $\bar{\mathrm{RMSE}}$ performance among the selected fusion models.

**Figure 12:**
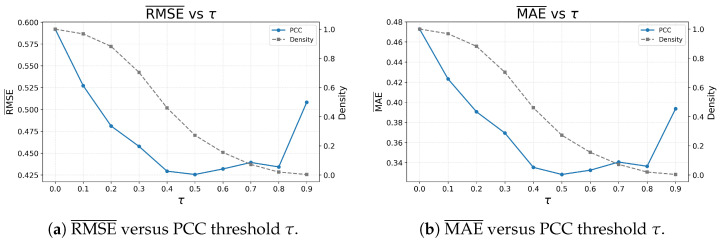
Sensitivity of GAT-based prediction error to the PCC threshold τ. The setting τ=0 denotes a fully connected graph. The blue curve shows prediction error, while the grey dashed curve shows graph density. As τ increases, density decreases because fewer landmark pairs satisfy the PCC threshold. The selected threshold τ=0.6 provides a favourable trade-off between prediction error and graph compactness while avoiding excessive fragmentation.

Figure 12 shows the effect of varying the PCC threshold τ on prediction error and graph structure. The evaluated settings include the fully connected graph, denoted by τ=0, and thresholded PCC graphs with τ∈{0.1,0.2,…,0.9}. In addition to $\bar{\mathrm{RMSE}}$ and $\bar{\mathrm{MAE}}$, the figure also shows the corresponding graph density, which decreases monotonically with increasing τ. The fully connected graph gives the highest error, with $\bar{\mathrm{RMSE}}$ 0.592 and $\bar{\mathrm{MAE}}$ 0.472, and has density close to 1, indicating that nearly all possible landmark pairs are connected. As τ increases from 0.1 to 0.5, both $\bar{\mathrm{RMSE}}$ and $\bar{\mathrm{MAE}}$ decrease substantially while density drops, suggesting that removing weakly correlated edges improves the usefulness of the graph topology. The lowest mean error is observed at τ=0.5, with $\bar{\mathrm{RMSE}}$ 0.426 and $\bar{\mathrm{MAE}}$ 0.328, using approximately 620 edges and no isolated nodes.

Although τ=0.5 gives the lowest numerical error, it also retains a denser topology than τ=0.6. This indicates that more moderate landmark associations are preserved, which may reduce interpretability and dilute the influence of the strongest landmark-to-landmark dependencies during message passing. In contrast, τ=0.6 retains only strongly correlated landmark pairs, reducing the graph to approximately 353 edges while maintaining comparable error, with $\bar{\mathrm{RMSE}}$ 0.432 and $\bar{\mathrm{MAE}}$ 0.332. This corresponds to a visibly lower density in Figure 12. Although τ=0.6 introduces about two isolated nodes on average, the overall graph remains sufficiently connected for message passing and provides a more compact and interpretable topology. Therefore, τ=0.6 is preferred as a balanced threshold rather than as the absolute lowest-error threshold.

This trade-off is important because the graph should provide a stable, interpretable structural prior rather than simply minimising error under a single validation setting. Lower thresholds, such as τ=0.1 and τ=0.2, retain more than 2000 edges and exhibit high density, making the graph close to fully connected and reducing the interpretability of landmark-specific dependencies. Conversely, higher thresholds increasingly fragment the graph. For example, τ=0.8 retains only about 45 edges and produces approximately 25.5 isolated nodes, while τ=0.9 retains only about 7 edges and produces approximately 55.8 isolated nodes. Although τ=0.8 still gives competitive error, the sharp reduction in density and the large number of isolated nodes weaken message passing and make the topology less suitable as a general landmark-dependency prior. Therefore, τ=0.6 is selected as the final threshold because it offers a favourable balance among $\bar{\mathrm{RMSE}}$, $\bar{\mathrm{MAE}}$, density, edge count, and isolated nodes.

Table 11 compares graph construction using Pearson, Spearman, and Kendall correlation coefficients at the selected threshold τ=0.6. Pearson provides the lowest prediction error, with $\bar{\mathrm{RMSE}}$ 0.432 and $\bar{\mathrm{MAE}}$ 0.332, followed by Spearman with $\bar{\mathrm{RMSE}}$ 0.438 and $\bar{\mathrm{MAE}}$ 0.339. Kendall produces higher error, with $\bar{\mathrm{RMSE}}$ 0.447 and $\bar{\mathrm{MAE}}$ 0.346. The graph statistics further explain this difference. Pearson and Spearman produce comparable edge counts, with approximately 353 and 334 edges, respectively, and both yield only about two isolated nodes on average. In contrast, Kendall produces a much sparser and more fragmented topology, with only about 53 edges and approximately 19.66 isolated nodes. The inference times are similar across the three correlation methods, indicating that the main difference lies not in computational overhead but in the quality and usability of the constructed topology. These results support the use of PCC for graph construction, as it yields the lowest prediction error while maintaining a sufficiently connected and interpretable landmark graph.

## 6. Conclusions and Future Works

This study proposed a graph–tabular latent-representation fusion framework for body temperature prediction from thermal facial landmark profiles. The framework derives two complementary representations from a single thermal landmark signal: a graph representation that captures landmark-to-landmark thermal dependencies, and a tabular representation that preserves global landmark-temperature patterns. By independently encoding these two views and fusing them at the latent level, the proposed model combines a relational inductive bias with global feature abstraction for non-contact body temperature prediction.

The results show that latent-level graph–tabular fusion improves over strong stand-alone graph and tabular baselines, especially under the MNAR-imputed setting, which reflects structured occlusion-driven missingness. Among the fused configurations, GATs + MLPs achieved the lowest MNAR-imputed $\bar{\mathrm{RMSE}}$ (0.296), improving over its GAT and MLPs baselines by approximately 6.4% and 7.1%, respectively. GraphSAGE + MLPs achieved the lowest MNAR-imputed $\bar{\mathrm{MAE}}$ (0.243), improving over its GraphSAGE and MLPs baselines by approximately 6.8% and 4.7%, respectively. Under MCAR-imputed evaluation, GINs + Linear-ReLU provided the strongest balance between accuracy and efficiency, achieving the best MCAR-imputed $\bar{\mathrm{RMSE}}$ and $\bar{\mathrm{MAE}}$ while maintaining the highest FPS among the selected top fused configurations.

The PCC-guided graph-construction analysis further supports the reliability of the proposed landmark topology. The threshold sensitivity experiment shows that τ=0.6 provides a favourable balance between prediction error, edge count, and isolated nodes, while the correlation-method control experiment supports using PCC over Spearman and Kendall for graph construction in the evaluated setting. These findings strengthen the methodological basis for using a shared PCC-derived topology while preserving subject-specific thermal node features in a multi-graph formulation.

The structured MNAR simulation also contributes to the evaluation protocol. Instead of relying solely on uniformly random landmark removal, the MNAR setting models missingness induced by head-pose visibility, glasses, and face-mask occlusion. Its landmark-wise missingness patterns were validated against real TFD68 occlusion annotations, supporting its use as a realistic and reproducible proxy for occlusion-driven missingness. This enables a more meaningful evaluation of model robustness under missing and imputed landmark-temperature conditions.

Statistical testing further confirms the benefit of fusion, particularly under MNAR-imputed evaluation. In the MNAR setting, significant improvements are observed in 75.0–77.1% of fused–baseline comparisons, depending on the metric and statistical test. Under MCAR-imputed evaluation, the proportion of significant improvements is lower (31.2–45.8%), indicating that fusion is especially beneficial when missingness is structured by pose and occlusion rather than randomly distributed. The remaining non-significant cases show that fusion effectiveness depends on the complementarity between graph and tabular backbones. Therefore, the proposed approach should be interpreted as a flexible design space rather than a universally optimal architecture.

From a practical perspective, the best MNAR-imputed fused configurations achieve $\bar{\mathrm{MAE}}$ values of approximately one-quarter of a degree Celsius while maintaining practical inference throughput. The accuracy-efficiency analyses indicate that different fused configurations serve different deployment priorities: GATs + MLPs is preferable when minimizing MNAR-imputed $\bar{\mathrm{RMSE}}$ is the main objective; GraphSAGE + MLPs provides the lowest MNAR-imputed $\bar{\mathrm{MAE}}$; and GINs + Linear-ReLU offers the highest throughput among the selected top configurations. These results suggest improved screening reliability under conditions of missing or imputed landmarks. The practical interpretation of these errors is based on the ISO-related accuracy assumption discussed in this study; therefore, the framework should be viewed as a modelling approach to improve landmark-based thermal prediction rather than a replacement for calibrated clinical thermometry.

Several directions remain for future work. Although TFD68 provides controlled landmark-temperature annotations, it is collected primarily from an Asian population; therefore, broader demographic validation is required to assess generalisability across population groups, skin characteristics, and acquisition settings. The obtained prediction errors are aligned with the ISO-based accuracy assumption used for non-contact thermographic temperature measurement in this study. Future work will compare eye-corner temperature estimates with contact-based body temperature measurements to more directly assess their suitability for predicting core body temperature. Methodologically, future work may explore data-adaptive topology learning, learnable adjacency estimation, or dynamic graph-structure optimisation to move beyond a fixed PCC-derived graph. Additional extensions include incorporating environmental and physiological covariates, modelling uncertainty in missing and imputed landmarks, and conducting prospective deployment studies that jointly assess prediction accuracy, throughput, and operational reliability in practical thermal-screening scenarios.

## Figures and Tables

**Table 1:** Comparison between real TFD68 occlusion annotations and the simulated MNAR masks. The TFD68 statistics are computed after excluding the challenging split and mixed or uncertain accessory cases.

Specifications	TFD68	Simulated MNAR
Pose configurations per participant	65	65
Landmarks per image	68	68
Occlusion scenarios	Self-occlusion, glasses, face mask	Self-occlusion, glasses, face mask
Minimum missingness percentage	0.00%	5.88%
Maximum missingness percentage	98.53%	86.76%
Mean missing landmarks per image	32.12	33.95
Median missing landmarks per image	33.00	36.00

**Table 2:** Agreement between real TFD68 occlusion distributions and simulated MNAR missingness masks. Agreement is computed from landmark-wise missing probabilities across the 65 pose configurations.

Agreement Metric	Self-Occlusion	Glasses	Face Mask
Landmark-wise Pearson correlation	0.981	0.852	0.845
Landmark-wise Spearman correlation	0.972	0.797	0.850
Landmark-wise MAE	0.020	0.104	0.107

**Table 3:** Graph-branch backbone architectures used in the fusion model. Default settings: graph hidden dimension $D_{h}=64$, number of graph layers L=3, and dropout p=0.3.

Backbone	Architecture
GraphSAGE	Input projection: Linear (4→64), BatchNorm, ReLU, Dropout. Message passing: 3× SAGEConv (64→64,aggr=mean), each followed by BatchNorm, ReLU, Dropout, and residual addition.
GCNs	Input projection: Linear (4→64), BatchNorm, ReLU, Dropout. Message passing: 3× GCNConv (64→64), each followed by BatchNorm, ReLU, Dropout, and residual addition.
GATs	Input projection: Linear (4→64), BatchNorm, ELU, Dropout. Message passing: 3× GATConv with 4 heads, per-head dimension 16, concatenated output 64, each followed by BatchNorm, ELU, Dropout, and residual addition.
GINs	Input projection: Linear (4→64), BatchNorm, ReLU, Dropout. Message passing: 3× GINConv, each with internal MLP 64→64→64 with ReLU, followed by BatchNorm, ReLU, Dropout, and residual addition.

**Table 4:** Tabular-branch backbone architectures used in the fusion model. Default settings: tabular input dimension 67, hidden dimension 128, output latent dimension 64, and dropout p=0.3 unless otherwise stated.

Backbone	Architecture
MLPs	Linear (67→128), ReLU, Dropout, Linear (128→64), ReLU.
CNN1D	Conv1d (1→32,k=3), ReLU; Conv1d (32→64,k=3), ReLU; AdaptiveAvgPool1d (1); Flatten; Dropout; Linear (64→64), ReLU.
Linear-ReLU	Linear (67→64), ReLU.
ResNet-style MLP	Input projection: Linear (67→128). Residual stack: 3× blocks with BatchNorm, Linear (128→256), ReLU, Dropout, Linear (256→128), Dropout, residual addition. Output: BatchNorm, ReLU, Linear (128→64), ReLU.
TabNet-style	BatchNorm (67); initial attention Linear (67→128) + ReLU; 4 decision steps, each with masker Linear (128→67) and transformer MLP (67→128→128) with ReLU, Dropout, and LayerNorm; final Linear (128→64), ReLU. Uses γ=1.3.
FT-Transformer	Feature tokenisation into 128-dimensional tokens; learned CLS token and column embeddings; 2 Transformer encoder layers with $d_{\mathrm{model}}=128$, 8 heads, feedforward dimension 256, GELU, Dropout, pre-layer normalisation; LayerNorm on CLS output; Linear (128→64), ReLU.

**Table 5:** Architectural summary of the stand-alone baseline models. The baselines are grouped by input modality: tabular models operate on the landmark-temperature vector, whereas graph models operate on graph-structured landmark inputs with node features formed as $\left[x_{i}∥c_{i}∥o_{i}\right]$. Each row specifies one unimodal reference architecture trained under the same supervised regression protocol and used to contextualise the proposed graph–tabular fusion models.

Group	Model	Architecture
Tabular	Linear-ReLU	Linear projection to 64 hidden units, ReLU, followed by a scalar head (64→64→1) with ReLU and dropout 0.3.
Tabular	MLPs	Encoder (67→128→64) with ReLU and dropout 0.3, followed by a scalar head (64→64→1).
Tabular	CNN1D	1D convolutions (1→32→64), adaptive average pooling, 64-dimensional projection, and scalar head (64→64→1).
Tabular	ResNet-style MLP	Input projection to 128 units, 3 residual blocks with expansion factor 2, batch normalisation, dropout 0.3, 64-dimensional projection, and scalar head.
Tabular	TabNet	Batch-normalised input, 4 sequential decision steps, hidden width 128, γ=1.3, 64-dimensional projection, and scalar head.
Tabular	FT-Transformer	Feature tokenisation into 128-dimensional tokens, 2 Transformer encoder layers, 8 attention heads, dropout 0.3, 64-dimensional projection, and scalar head.
Graph	GCNs	Augmented node input, linear projection (4→64), 3 residual GCN layers, batch normalisation, ReLU, dropout 0.3, and node-wise linear output head.
Graph	GraphSAGE	Augmented node input, linear projection (4→64), 3 residual GraphSAGE layers with mean aggregation, batch normalisation, ReLU, dropout 0.3, and node-wise linear output head.
Graph	GATs	Augmented node input, linear projection (4→64), 3 residual 4-head graph attention layers, ELU, dropout 0.3, and node-wise linear output head.
Graph	GINs	Augmented node input, linear projection (4→64), 3 residual GINs layers with internal MLPs (64→64→64), ReLU, dropout 0.3, and node-wise linear output head.

**Table 6:** Prediction error and standard deviations for baseline models under Complete, Missing, and Imputed conditions using CMILK. Best values in each column are highlighted in bold.

Method	Complete		Missing								Imputed							
			MNAR				MCAR				MNAR				MCAR			
	RMSE	MAE	$\bar{\mathrm{RMSE}}$	$\bar{\mathrm{MAE}}$	$\sigma_{R}$	$\sigma_{M}$	$\bar{\mathrm{RMSE}}$	$\bar{\mathrm{MAE}}$	$\sigma_{R}$	$\sigma_{M}$	$\bar{\mathrm{RMSE}}$	$\bar{\mathrm{MAE}}$	$\sigma_{R}$	$\sigma_{M}$	$\bar{\mathrm{RMSE}}$	$\bar{\mathrm{MAE}}$	$\sigma_{R}$	$\sigma_{M}$
Linear-ReLU	0.229	0.194	31.430	27.632	2.551	4.262	23.436	17.006	8.263	9.615	0.317	**0.254**	0.058	0.049	0.284	0.234	0.075	0.067
MLPs	0.226	0.188	32.495	28.846	2.630	4.263	24.211	17.877	8.648	9.893	0.319	0.255	0.059	0.050	0.284	0.233	0.077	0.069
CNN	0.347	0.278	32.931	28.924	2.688	4.500	24.444	18.022	9.002	10.490	0.373	0.312	0.063	0.058	0.373	0.305	0.042	0.038
ResNet-MLP	**0.225**	0.194	31.820	28.327	2.483	4.053	23.587	17.225	8.216	9.474	0.364	0.285	0.075	0.063	0.283	0.230	0.065	0.046
TabNet	0.225	**0.184**	31.091	27.209	2.482	4.259	23.025	16.717	8.149	9.600	0.332	0.256	0.067	0.054	0.289	0.234	0.101	0.089
FT-Transformer	0.245	0.198	31.706	27.674	2.495	4.342	23.344	16.746	8.245	9.749	0.348	0.267	0.071	0.057	0.313	0.250	0.120	0.110
GCNs	0.257	0.223	1.586	1.423	0.101	0.145	1.386	1.265	0.411	0.445	0.319	0.263	0.032	0.028	0.270	0.231	0.041	0.033
GraphSAGE	0.255	0.221	3.192	2.805	0.227	0.346	2.268	2.127	1.118	1.164	0.315	0.260	0.029	0.027	0.270	0.229	0.041	0.033
GATs	0.262	0.219	**1.356**	**1.119**	**0.067**	**0.087**	**0.865**	**0.766**	**0.341**	**0.320**	0.317	0.259	**0.022**	**0.022**	0.281	0.234	**0.039**	**0.032**
GINs	0.249	0.213	4.530	3.863	0.372	0.561	2.892	2.686	1.838	1.890	**0.310**	0.256	0.026	0.024	**0.268**	**0.226**	0.042	0.034

**Table 7:** Training time (TT), inference time (IT), and frames per second (FPS) for baseline models under Complete, Missing, and Imputed conditions using CMILK. Best values in each column are highlighted in bold.

Method	TT (s)	Complete		Missing				Imputed			
				MNAR		MCAR		MNAR		MCAR	
		IT (s)	FPS	IT (s)	FPS	IT (s)	FPS	IT (s)	FPS	IT (s)	FPS
Linear-ReLU	**97.828**	0.187	5.348	**0.146**	**6.849**	**0.142**	**7.042**	**0.145**	**6.897**	**0.141**	**7.092**
MLPs	116.281	**0.162**	**6.173**	0.162	6.173	0.159	6.289	0.165	6.061	0.162	6.173
CNN	149.694	0.225	4.444	0.196	5.102	0.202	4.950	0.195	5.128	0.199	5.025
ResNet-MLP	255.422	0.344	2.907	0.302	3.311	0.297	3.367	0.300	3.333	0.295	3.390
TabNet	432.369	0.480	2.083	0.488	2.049	0.499	2.004	0.489	2.045	0.489	2.045
FT-Transformer	404.505	0.388	2.577	0.310	3.226	0.310	3.226	0.311	3.215	0.310	3.226
GCNs	1243.198	1.587	0.630	1.695	0.590	1.708	0.585	1.690	0.592	1.728	0.579
GraphSAGE	917.939	1.332	0.751	1.361	0.735	1.354	0.739	1.352	0.740	1.343	0.745
GATs	1627.387	2.038	0.491	1.931	0.518	1.926	0.519	1.936	0.517	1.931	0.518
GINs	1042.571	1.399	0.715	1.326	0.754	1.325	0.755	1.358	0.736	1.323	0.756

**Table 8:** Prediction error and standard deviations for fused graph–tabular models under Complete, Missing, and Imputed conditions using CMILK. Best values in each column are highlighted in bold.

Graph	Tabular	Complete		Missing								Imputed							
				MNAR				MCAR				MNAR				MCAR			
		RMSE	MAE	$\bar{\mathrm{RMSE}}$	$\bar{\mathrm{MAE}}$	$\sigma_{R}$	$\sigma_{M}$	$\bar{\mathrm{RMSE}}$	$\bar{\mathrm{MAE}}$	$\sigma_{R}$	$\sigma_{M}$	$\bar{\mathrm{RMSE}}$	$\bar{\mathrm{MAE}}$	$\sigma_{R}$	$\sigma_{M}$	$\bar{\mathrm{RMSE}}$	$\bar{\mathrm{MAE}}$	$\sigma_{R}$	$\sigma_{M}$
GATs	CNN1D	0.255	0.219	0.436	0.330	**0.013**	0.019	0.397	0.308	**0.029**	**0.022**	0.309	0.257	0.019	0.018	0.275	0.232	0.037	0.026
GATs	FT-Transformer	0.243	0.211	**0.427**	**0.322**	0.013	**0.018**	**0.371**	**0.291**	0.037	0.028	0.305	0.254	0.022	0.021	0.267	0.227	0.041	0.033
GATs	Linear-ReLU	0.212	0.168	5.959	5.306	0.373	0.479	5.417	5.296	2.787	2.904	0.300	0.244	0.030	0.028	0.248	0.206	0.055	0.052
GATs	MLPs	0.222	0.188	4.618	4.194	0.315	0.387	4.176	4.104	2.193	2.257	**0.296**	0.245	0.025	0.023	0.251	0.211	0.048	0.041
GATs	ResNet-MLP	0.216	0.182	1.681	1.112	0.533	0.294	0.962	0.701	0.361	0.196	0.308	0.251	0.027	0.024	0.256	0.215	0.059	0.050
GATs	TabNet	0.230	0.193	0.552	0.424	0.024	0.024	0.485	0.369	0.094	0.074	0.326	0.267	0.032	0.027	0.268	0.228	0.059	0.052
GINs	CNN1D	0.269	0.227	0.482	0.395	0.032	0.031	0.599	0.501	0.145	0.152	0.314	0.259	0.017	0.016	0.287	0.238	0.035	0.026
GINs	FT-Transformer	0.257	0.219	0.443	0.352	0.022	0.021	0.497	0.406	0.097	0.101	0.309	0.255	0.019	0.018	0.278	0.232	0.037	0.031
GINs	Linear-ReLU	0.213	0.171	5.539	4.906	0.345	0.444	5.044	4.920	2.611	2.727	0.298	0.244	0.029	0.027	**0.247**	0.206	0.055	0.051
GINs	MLPs	0.230	0.193	4.323	3.947	0.310	0.369	3.953	3.874	2.166	2.234	0.298	0.246	0.023	0.021	0.256	0.215	0.046	0.039
GINs	ResNet-MLP	0.216	0.183	1.663	0.996	0.589	0.318	0.888	0.629	0.399	0.237	0.306	0.250	0.026	0.024	0.255	0.214	0.057	0.048
GINs	TabNet	0.231	0.193	0.530	0.404	0.023	0.021	0.477	0.359	0.061	0.041	0.325	0.267	0.031	0.027	0.267	0.227	0.058	0.051
GCNs	CNN1D	0.264	0.224	0.459	0.358	0.022	0.022	0.493	0.381	0.039	0.027	0.313	0.258	0.018	0.017	0.283	0.235	0.036	0.026
GCNs	FT-Transformer	0.257	0.219	0.463	0.348	0.021	0.021	0.483	0.366	0.030	0.025	0.309	0.255	0.019	0.018	0.277	0.231	0.038	0.030
GCNs	Linear-ReLU	0.215	0.173	5.439	4.831	0.338	0.436	4.966	4.849	2.590	2.700	0.298	0.243	0.028	0.026	0.247	0.206	0.054	0.050
GCNs	MLPs	0.230	0.194	4.245	3.866	0.293	0.356	3.763	3.687	2.077	2.141	0.299	0.246	0.022	0.021	0.257	0.215	0.046	0.039
GCNs	ResNet-MLP	**0.210**	0.176	1.456	0.878	0.510	0.275	0.836	0.590	0.366	0.209	0.304	0.247	0.027	0.024	0.251	0.210	0.059	0.050
GCNs	TabNet	0.232	0.194	0.482	0.362	0.023	0.021	0.455	0.339	0.049	0.031	0.324	0.266	0.030	0.026	0.267	0.227	0.057	0.050
GraphSAGE	CNN1D	0.278	0.234	0.547	0.455	0.036	0.036	0.520	0.424	0.092	0.105	0.318	0.261	**0.015**	**0.014**	0.295	0.244	**0.032**	**0.022**
GraphSAGE	FT-Transformer	0.260	0.220	0.439	0.348	0.020	0.020	0.427	0.331	0.031	0.039	0.311	0.256	0.018	0.017	0.281	0.234	0.037	0.031
GraphSAGE	Linear-ReLU	0.212	**0.167**	4.847	4.250	0.296	0.364	4.339	4.199	2.168	2.293	0.300	0.244	0.030	0.027	0.247	**0.206**	0.055	0.052
GraphSAGE	MLPs	0.223	0.185	4.112	3.720	0.251	0.309	3.659	3.558	1.872	1.961	0.297	**0.243**	0.024	0.022	0.252	0.210	0.049	0.043
GraphSAGE	ResNet-MLP	0.224	0.187	1.723	1.010	0.609	0.342	1.006	0.700	0.378	0.167	0.313	0.254	0.025	0.022	0.262	0.218	0.056	0.045
GraphSAGE	TabNet	0.231	0.193	0.503	0.380	0.022	0.019	0.471	0.354	0.063	0.044	0.323	0.265	0.030	0.026	0.267	0.227	0.056	0.049

**Table 9:** Training time (TT), inference time (IT), and frames per second (FPS) for fused graph–tabular models under Complete, Missing, and Imputed conditions using CMILK. Best values in each column are highlighted in bold.

Graph	Tabular	TT (s)	Complete		Missing				Imputed			
					MNAR		MCAR		MNAR		MCAR	
			IT (s)	FPS	IT (s)	FPS	IT (s)	FPS	IT (s)	FPS	IT (s)	FPS
GATs	CNN1D	1446.924	3.418	0.293	3.442	0.290	3.444	0.290	3.437	0.291	3.446	0.290
GATs	FT-Transformer	1508.950	4.224	0.237	4.419	0.226	4.397	0.227	4.375	0.229	4.367	0.229
GATs	Linear-ReLU	1480.017	3.328	0.300	3.317	0.301	3.312	0.302	3.325	0.301	3.333	0.300
GATs	MLPs	1459.675	3.443	0.290	3.347	0.299	3.353	0.298	3.525	0.284	3.358	0.298
GATs	ResNet-MLP	1496.444	3.626	0.276	3.622	0.276	3.599	0.278	3.614	0.277	3.587	0.279
GATs	TabNet	1585.743	3.922	0.255	3.904	0.256	3.916	0.255	3.900	0.256	3.927	0.255
GINs	CNN1D	**926.955**	2.972	0.336	2.939	0.340	2.955	0.338	2.959	0.338	2.953	0.339
GINs	FT-Transformer	1105.582	3.549	0.282	4.094	0.244	3.608	0.277	4.114	0.243	3.727	0.268
GINs	Linear-ReLU	1080.274	2.926	0.342	**2.809**	**0.356**	2.791	0.358	2.822	0.354	2.836	0.353
GINs	MLPs	978.554	2.944	0.340	2.883	0.347	2.905	0.344	2.878	0.347	2.841	0.352
GINs	ResNet-MLP	1091.330	3.109	0.322	3.141	0.318	3.124	0.320	3.133	0.319	3.153	0.317
GINs	TabNet	1169.300	3.422	0.292	3.420	0.292	3.532	0.283	3.428	0.292	3.446	0.290
GCNs	CNN1D	1048.890	3.212	0.311	3.205	0.312	3.213	0.311	3.208	0.312	3.214	0.311
GCNs	FT-Transformer	1213.271	3.837	0.261	4.015	0.249	4.033	0.248	4.013	0.249	4.016	0.249
GCNs	Linear-ReLU	1140.910	3.185	0.314	3.093	0.323	3.093	0.323	3.104	0.322	3.139	0.319
GCNs	MLPs	1116.946	3.200	0.313	3.124	0.320	3.123	0.320	3.127	0.320	3.129	0.320
GCNs	ResNet-MLP	1123.092	3.501	0.286	3.388	0.295	3.396	0.294	3.391	0.295	3.390	0.295
GCNs	TabNet	1220.579	3.758	0.266	3.718	0.269	3.730	0.268	3.699	0.270	3.739	0.267
GraphSAGE	CNN1D	1099.957	3.117	0.321	2.933	0.341	2.939	0.340	2.960	0.338	2.944	0.340
GraphSAGE	FT-Transformer	1217.726	3.463	0.289	3.651	0.274	3.620	0.276	3.693	0.271	3.653	0.274
GraphSAGE	Linear-ReLU	1209.172	2.946	0.339	2.819	0.355	**2.790**	**0.358**	**2.792**	**0.358**	**2.801**	**0.357**
GraphSAGE	MLPs	1124.803	**2.829**	**0.353**	2.839	0.352	2.823	0.354	2.851	0.351	2.830	0.353
GraphSAGE	ResNet-MLP	1076.980	3.219	0.311	3.155	0.317	3.135	0.319	3.161	0.316	3.187	0.314
GraphSAGE	TabNet	1160.107	3.390	0.295	3.434	0.291	3.428	0.292	3.424	0.292	3.442	0.291

**Table 10:** Paired statistical comparison between selected fused graph–tabular models and their corresponding graph-only or tabular-backbone baselines under the MNAR-imputed condition. Positive Cohen’s $d_{z}$ indicates that the fused model reduced error relative to the compared baseline.

Fused Graph–Tabular	Graph	Tabular	$\bar{\mathrm{RMSE}}$				$\bar{\mathrm{MAE}}$			
			t-Value	t-p	Wilcoxon-p	Cohen’s $d_{z}$	t-Value	t-p	Wilcoxon-p	Cohen’s $d_{z}$
GATs + MLPs	GATs	–	34.53	$8.62\times 10^{-85}$	$9.48\times 10^{-34}$	2.47	25.64	$3.15\times 10^{-64}$	$6.26\times 10^{-33}$	1.84
GATs + MLPs	–	MLPs	5.12	$7.24\times 10^{-7}$	$9.74\times 10^{-7}$	0.37	2.87	$4.52\times 10^{-3}$	$4.62\times 10^{-3}$	0.21
GraphSAGE + MLPs	GraphSAGE	–	19.41	$2.51\times 10^{-47}$	$2.77\times 10^{-32}$	1.39	23.54	$9.16\times 10^{-59}$	$3.20\times 10^{-33}$	1.69
GraphSAGE + MLPs	–	MLPs	5.02	$1.16\times 10^{-6}$	$1.74\times 10^{-6}$	0.36	3.32	$1.06\times 10^{-3}$	$1.32\times 10^{-3}$	0.24
GINs + Linear-ReLU	GINs	–	16.04	$2.08\times 10^{-37}$	$3.65\times 10^{-27}$	1.15	19.11	$1.75\times 10^{-46}$	$6.92\times 10^{-30}$	1.37
GINs + Linear-ReLU	–	Linear-ReLU	4.18	$4.41\times 10^{-5}$	$5.37\times 10^{-5}$	0.30	2.82	$5.24\times 10^{-3}$	$5.06\times 10^{-3}$	0.20

**Table 11:** Control experiment comparing correlation methods for graph construction at τ=0.6. All models are trained under the same GAT-based graph-only validation setting and hyperparameters.

Correlation	$\bar{\mathrm{RMSE}}$	$\bar{\mathrm{MAE}}$	Edges	Isolated Nodes	Infer. Time
Pearson	0.432	0.332	352.821	2.000	0.340
Spearman	0.438	0.339	334.242	1.979	0.335
Kendall	0.447	0.346	52.600	19.663	0.331

## Data Availability

The dataset, code, and pre-trained models supporting this study are publicly available at: https://github.com/lucas-nyc/GTLF_BTP (accessed on 1 May 2026).
